# Magneto-Fluorescent Microrobots with Selective Detection Intelligence for High-Energy Explosives and Antibiotics in Aqueous Environments

**DOI:** 10.1021/acsami.5c02259

**Published:** 2025-03-27

**Authors:** N. Senthilnathan, Cagatay M. Oral, Martin Pumera

**Affiliations:** † Future Energy and Innovation Laboratory, Central European Institute of Technology, Brno University of Technology, Purkynova 123, Brno 61200, Czech Republic; ‡ Advanced Nanorobots & Multiscale Robotics Laboratory, Faculty of Electrical Engineering and Computer Science, VSB—Technical University of Ostrava, 17. listopadu 2172/15, Ostrava 70800, Czech Republic; § Department of Medical Research, China Medical University Hospital, China Medical University, No. 91 Hsueh-Shih Road, Taichung 40402, Taiwan

**Keywords:** magnetic microrobots, organic pollutants, environmental monitoring, charge transfer complexes, fluorescence sensing

## Abstract

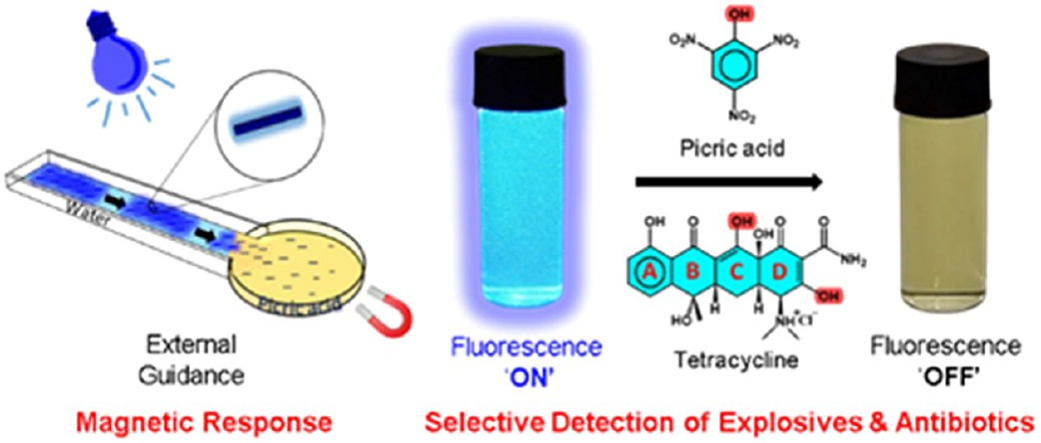

Fluorescence-based sensing is a straightforward and powerful technique with high sensitivity for the detection of a wide range of chemical and biological analytes. Integrating the high sensing capabilities of fluorescent probes with wireless navigation systems can enable the extension of their operational range, even in challenging scenarios with limited accessibility or involving hazardous substances. This study presents the development of molecularly engineered magneto-fluorescent microrobots based on the push–pull quinonoids by incorporating magnetic nanoparticles using a reprecipitation approach with the aim of detecting high-energy explosives and antibiotics in aqueous environments. The magnetic components in the microrobots offer remotely controlled navigability toward the intended target areas under the guidance of external magnetic fields. Upon interactions with either explosives (picric acid) or antibiotics (tetracycline), the microrobots’ intrinsic fluorescence switches to a “fluorescence off” state, enabling material-based intelligence for sensing applications. The molecular-level interactions that underlie “on–off” fluorescence state switching upon engagement with target molecules are elucidated through extensive spectroscopy, microscopy, and X-ray diffraction analyses. The microrobots’ selectivity toward target molecules is achieved by designing microrobots with amine functionalities capable of intermolecular hydrogen bonding with the acidic hydroxyl group of picric acid, leading to the formation of water-soluble charge transfer picrate complexes through proton transfer. Similarly, proton transfer interactions play a key role in tetracycline detection. The selective fluorescence switching performance of microrobots in fluidic channel experiments illustrates their selective sensing intelligence for target molecules in an externally controlled manner, highlighting their promising characteristics for sensing applications in real-world scenarios.

## 1 Introduction

Advanced explosives detection strategies are continuously being developed to neutralize the increasing threat of militant attacks in the modern era. Despite developments in this field, more sophisticated detection techniques should be devised for a peaceful and secure future. Picric acid (2,4,6-trinitrophenol) is a highly explosive compound among the other nitroaromatic explosives due to its strong acidity and high tendency to react with metals, thanks to all three electron-donating nitro groups.^1−3^ Moreover, picric acid can cause severe adverse effects on the skin and eyes and damage the vital organs of the body, including kidneys, liver, and lungs.^4,5^ Apart from being a powerful explosive, picric acid is also a potential environmental pollutant because of its diverse utilization in different manufacturing industries, including dyes, pharmaceuticals, and insecticides.^6−8^ Among the existing methodologies and fluorescent probes for the detection of picric acid, including metal–organic frameworks (MOFs),^9^ conjugated polymers,^10^ quantum dots^11,12^ and nanoparticles,^13^ probes based on fluorescent small organic molecules can be considered as attractive candidates due to their nontoxicity, eco-friendly, and high structural tailorability.^14,15^ However, developing a new class of probes based on fluorescent small organic molecules with aggregation-induced emission (AIE) for the selective detection of picric acid in the presence of other nitroaromatic explosives is a significant challenge.^16^

Since its discovery as an antibiotic, tetracycline has been widely used for its excellent antibacterial activity.^17^ However, its overexploitation causes antibacterial resistance in humans as well as animals,^18^ and contaminates the environment as it does not fully break down under ambient conditions.^19^ Consequently, significant quantities of tetracycline are excreted into the soil and water bodies,^20^ leading to the contamination of even animal-derived food products such as meat, milk, and egg.^21^ As per the European Union regulations, traces of tetracycline should not exceed more than 200 and 100 μg/kg for egg and chicken, respectively.^22^ Therefore, the development of detection methodologies for tetracycline in water bodies is essential for environmental monitoring and its subsequent remediation. Generally, classical detection methods like electrochemical,^23^ calorimetric,^24^ fluorescent,^25^ and liquid chromatography^26^ have been suggested to detect tetracycline in aqueous samples. Of these, fluorescence methods and probes utilized for detection, including fluorescent hydrogels, fluorescent polymers, and fluorescent metal–organic complexes, stand out due to their high sensitivity and selectivity.^27−29^ However, probes based on fluorescent small organic molecules for the detection of tetracycline are not common. In general, most of the existing detection probes perform well only under defined laboratory conditions, but they have not been studied in complicated real-world scenarios.

In recent years, interest in micro/nanorobots has been continuously increasing in various fields due to their advanced locomotion features that enable them to complete defined tasks in target areas via an externally controllable manner.^30−32^ Among the microrobots that could propel under different external stimuli, like light,^33−35^ magnetic fields,^36−38^ electric field,^39,40^ and acoustic waves,^41^ magnetically driven microrobots are well suited for applications requiring long-range locomotion capabilities, including biomedical applications and environmental remediation.^42,43^ Our research group has developed several light-, magnetic-, and chemically powered microrobots for a range of biomedical and environmental applications, including cancer therapy,^44^ biofilm eradication,^45^ and the removal of bacterial cells^36^ and various small organic pollutants from water.^46,47^ Apart from these research directions, the photodegradation of nitroaromatic explosives and antibiotics has also been a subject of research.^48−52^ However, the detection of these contaminants via microrobots has not been investigated in detail previously. In this work, push–pull quinonoid-based magnetically driven fluorescent microrobots were developed to selectively detect picric acid from other nitroaromatic explosives and tetracycline in remote locations. A derivative of diaminodicyanoquinodimethane (DADQ) was chosen for the fabrication of molecular material-based microrobots due to its excellent solid-state fluorescence over their solution-state and fluorescence switching properties under different conditions.^53,54^ Their excellent structural tailorability allows us to engineer derivatives with the required functionalities for efficient interactions with target molecules in aqueous environments. In addition, previous research articles on DADQs have shown that they are nontoxic, which is critical for environmental monitoring applications.^55,56^

**Scheme 1:**
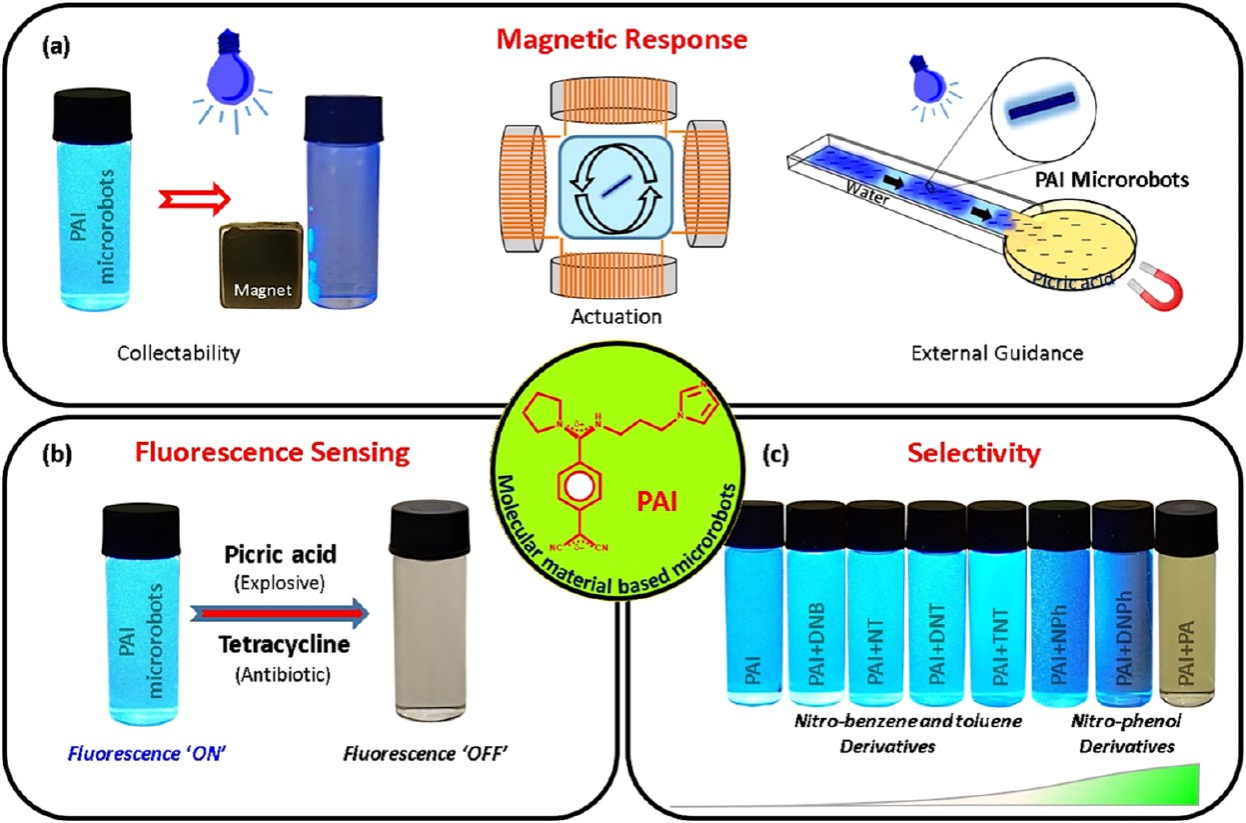
Schematic Illustration for Fluorescence Sensing Capabilities of PAI Microrobots toward Picric Acid and Tetracycline (a) Magnetic collectability, actuation, and external guidance of microrobots in the fluidic channel under the influence of external magnetic fields. (b) Fluorescence sensing of microrobots for picric acid and tetracycline. (c) Selective fluorescence sensing capabilities of microrobots for picric acid in the presence of various nitroaromatic explosives.

Specifically in this study, “(7-pyrrolidino-7-(1-(3-aminopropyl)imidazole)-8,8-dicyanoquinodimethane (PAI) was synthesized for the fabrication of molecular materials-based microrobots. In the structural composition of the microrobots, PAI molecules serve as the major structural components, while Fe_3_O_4_ nanoparticles are the minor partners. Here, Fe_3_O_4_ nanoparticles are employed to magnetize microrobots to facilitate their locomotion under the influence of external magnetic fields, providing remote navigability and collectability. Furthermore, various microscopic and spectroscopic analyses were employed to gain insight into the molecular-level changes as well as morphological modifications of PAI microrobots upon exposure to target analytes, i.e., picric acid and tetracycline. Combining the fluorescence sensing ability with remotely guided locomotion (Scheme 1) makes the microrobots more competent for sensing explosives and antibiotic molecules in real-world conditions. In summary, this study demonstrates the development of magnetically driven fluorescent microrobots with material-based intelligence for sensing organic pollutants in the aqueous environment.

## 2 Results and Discussion

### 2.1 Synthesis, Fabrication, and Characterization

**Figure 1:**
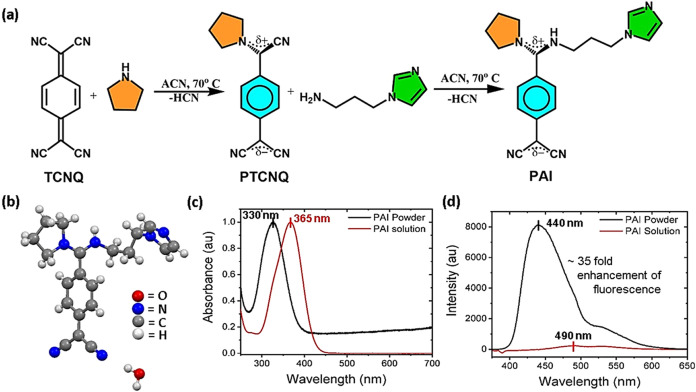
Synthesis and characterizations of 7-pyrrolidino-7-(1-(3-aminopropyl)imidazole)-8,8-dicyanoquinodimethane (PAI). (a) Scheme for the two-step synthesis of PAI. (b) Molecular structure of PAI molecule from single-crystal X-ray diffraction analysis. (c) Electronic absorption and (d) fluorescence emission spectra (*E*_x_: 350 nm) of PAI molecules in both solid and solution states.

A push–pull quinonoid-based PAI derivative was synthesized by substituting 1-(3-aminopropyl)imidazole into the quinodimethane framework of DADQ through a two-step synthetic route as shown in Figure 1a. Following purification, PAI was structurally characterized by employing single-crystal X-ray diffraction (SCXRD, Figure 1b), and nuclear magnetic resonance (NMR) spectroscopic analyses (Figure S1). PAI single crystals, cultivated from its acetonitrile solution, belong to a monoclinic crystal system with a space group of *P*2_1_/*n*. Their molecular structure, unit cell packing, and supramolecular assembly are shown in Figures 1b and S2. The basic crystal data are summarized in Table S1. In addition, mass spectroscopic analysis also confirms the formation of PAI molecules and their purity (Figure S3). Electronic absorption and fluorescence emission spectra of PAI in both solid and solution states are presented in Figure 1c,d, respectively. In the electronic absorption spectra, PAI molecules exhibit absorption maxima at 330 and 365 nm in their solid and solution states, respectively. The blue shift observed in the solid state is due to the push–pull molecular environments, and the rise of the absorption spectra can be resulted from intramolecular charge transfer in PAI molecules.^57^ PAI molecules exhibit fluorescence emission maxima at 450 and 490 nm in their solid and solution states, respectively, as shown in Figure 1d. It is interesting to note that PAI molecules not only show a blue shift in their solid state but also emit ≈35 times more fluorescence than their solution state, and this enhancement is likely due to the consequences of obstructions in the intra- and intermolecular nonradiative excited state energy decay.^58^ The fluorescence enhancement noticed in their solid state over the solution state is systematically analyzed by recording the emission spectra of PAI molecules in a series of solvent mixtures containing water and DMSO with increasing water fractions (Figure S4). The emission of PAI molecules shows significant enhancement when the water fraction is more than 0.6 in DMSO/water mixtures. This enhancement is attributed to the aggregation of PAI molecules, and it is popularly known as “aggregation-induced emission” (AIE),^59^ valuable for a wide range of applications, including biomedical and environmental monitoring.^60,61^

**Figure 2:**
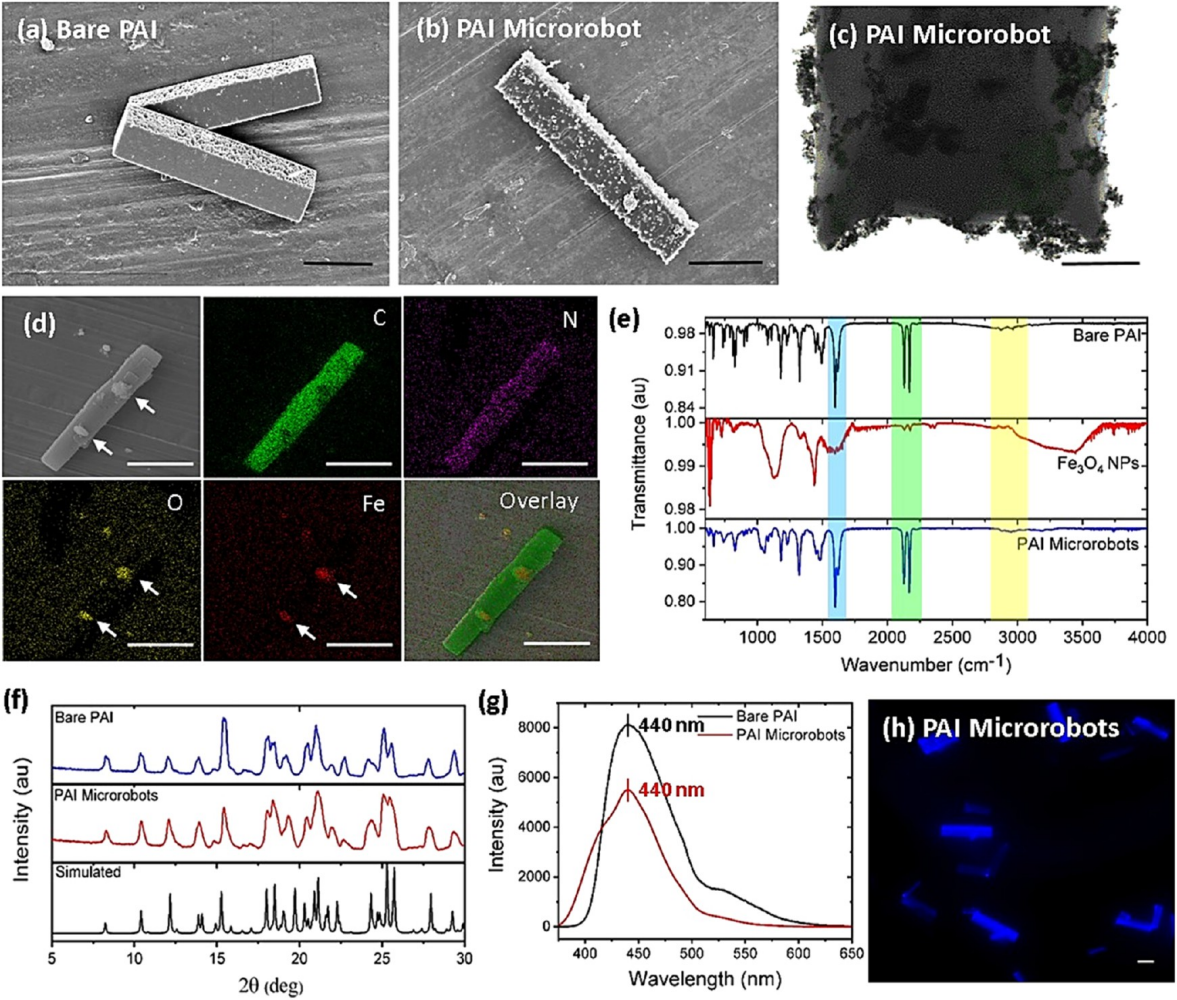
Characterization of PAI microrobots. FESEM images of (a) bare PAI microrods and (b) PAI microrobots (scale bars: 5 μm). (c) STEM image of a PAI microrobot having a decoration of Fe_3_O_4_ nanoparticles (scale bar: 500 nm). (d) EDAX elemental mapping (C, N, O, Fe, and overlay) of a PAI microrobot (scale bar: 10 μm). White arrows indicate clusters of Fe_3_O_4_ nanoparticles. (e) FTIR and (f) PXRD spectra of bare PAI microrods, Fe_3_O_4_ nanoparticles, and PAI microrobots. Simulated PXRD spectra of PAI crystals derived from their single-crystal X-ray diffraction analysis are shown for reference. (g) Fluorescence emission spectra (*E*_x_: 350 nm) of bare PAI and PAI microrobots. (h) Fluorescence micrograph of PAI microrobots (scale bar: 10 μm).

A simple reprecipitation method was adopted to decorate the surface of bare PAI microparticles with the Fe_3_O_4_ nanoparticles as shown in Figure S5. The addition of Fe_3_O_4_ nanoparticles magnetizes the bare PAI microparticles, which allows guidance toward the place of interest under external magnetic fields. According to field emission scanning electron microscopy (FESEM) analysis, the size of the microrobots is in the range of 10–15 μm. The similar morphology of the bare microrods (Figure 2a) and microrobots (Figure 2b) fabricated from PAI molecules illustrates that the incorporation of Fe_3_O_4_ nanoparticles during the fabrication process does not significantly influence the morphological features. Furthermore, scanning transmission electron microscopy (STEM) analysis exhibits the successful incorporation of Fe_3_O_4_ nanoparticles on the surface of the microrods (Figure 2c). Furthermore, elemental dispersive X-ray (EDAX) analysis confirms that the primary building block of microrobots comprises C atoms and the successful attachment of Fe_3_O_4_ nanoparticles on the surface of the PAI microrods (Figure 2d). The significant presence of magnetite nanoparticles is evident from the presence of Fe and O in the EDAX analysis. Fourier-transform infrared (FTIR, Figure 2e) and powder X-ray diffraction (PXRD, Figures 2f and S6a) studies exhibit that the characteristic peaks and peak positions of bare PAI microrods match with those of PAI microrobots. FTIR characteristic peaks of PAI compounds observed at 2941 (N–H), 2170 (conjugated nitrile), 2133 (conjugated nitrile), and 1595 cm^–1^ (C = C stretching of the benzene ring) reflect their structural integrity. PXRD spectra of both bare microrods and microrobots exhibit their crystallinity, proving once again that as expected the fabrication process does not change the crystallinity of PAI microrobots. The peaks of simulated powder XRD spectra of PAI molecules obtained from SCXRD analysis match with those of their measured spectra, as shown in Figure 2f. Fluorescence emission spectra of both bare PAI crystalline solids and their respective microrobots (Figure 2g) show that their emission maximum is not changed, suggesting unmodified photophysical properties of PAI molecules in both bare microrods and microrobots. Furthermore, the fluorescence micrograph of PAI microrobots in Figure 2h shows the blue fluorescence emission, which is consistent with their fluorescence emission spectra. Notably, the characteristic peaks of Fe_3_O_4_ nanoparticles are not observed in either the FTIR or PXRD spectra of PAI microrobots potentially because of their limited presence compared to PAI molecules, i.e., the microrobots’ building block.

### 2.2 Magnetic Actuation Studies

**Figure 3:**
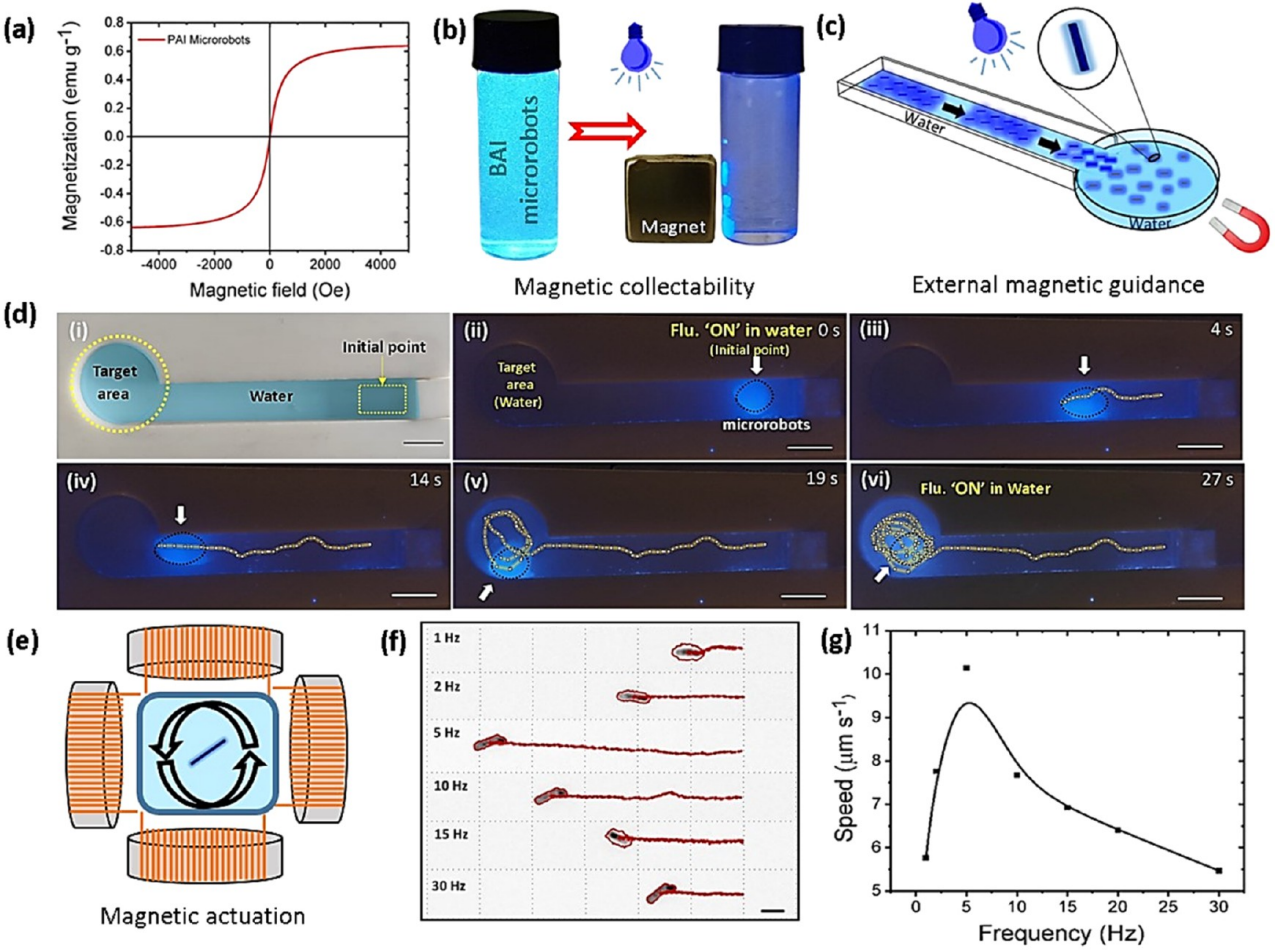
Magnetic actuation of microrobots. (a) Magnetic hysteresis loop of PAI microrobots. (b) Magnetic collectability of PAI microrobots under the influence of a permanent magnet. (c) Pictorial representation of a fluidic channel used in this study. (d) Time-lapse images of externally guided locomotion of the microrobots to the target area in the fluidic channel. Images were captured under (i) visible light and (ii–vi) UV-light illumination (scale bars: 1 cm). (e) Pictorial illustration of a custom-built magnetic setup used for magnetic actuation experiments in this study. (f) Displacement (scale bar: 10 μm) and (g) average speed of the microrobots in the frequency range of 1 to 30 Hz at 5 mT.

Vibrating sample magnetometer (VSM) analysis was carried out to comprehend the magnetic properties of PAI microrobots (Figure 3a) and Fe_3_O_4_ nanoparticles (Figure S6b). The magnetic hysteresis loops reveal that the incorporation of superparamagnetic Fe_3_O_4_ nanoparticles can turn bare PAI microrods into magnetic microrobots, which can facilitate their magnetic locomotion toward far-off target molecules for sensing. By combining magnetic collectability and externally guided motion to fluorescence sensing capabilities, fluorescent probes can be adapted for sensing applications in real-world scenarios. The magnetic collectability of the microrobots was examined using a permanent neodymium–iron–boron (NdFeB) magnet. In the experiment, the magnet was simply placed near the vial filled with microrobots, which caused them to be collected near the magnet, and images of the vials were captured under the illumination of ultraviolet (UV)-light (Figure 3b), illustrating their efficient magnetic collectability. In addition, the externally guided motion of the microrobots under the influence of external magnetic fields was tested by employing a fluidic channel (its pictorial representation is shown in Figure 3c). The photograph of the fluidic channel used in our experiments is shown in Figure 3d(i). A few drops of magneto-fluorescent microrobots were added as fluorescence “ON” state at the initial point of the fluidic channel while the target area was filled with water. Then, the microrobots were directed toward the target area and navigated circularly for several rounds under the influence of the permanent magnet, indicating the externally target-guided navigation capability of the microrobots (Figure 3d(ii–vi)). The fluorescence response of the microrobots was monitored throughout the entire experiment under UV-light illumination, and their time-lapse images were captured (Movie S1). Notably, the fluorescence “ON” state did not change throughout the experiment, as expected.

In addition to magnetic collectability and external guidance, the magnetic controllability of the microrobots was analyzed by employing a custom-built magnetic setup coupled with an optical microscope and an advanced controlling unit (Figure 3e). The magnetic setup customized with three orthogonal coil pairs was employed to generate transversal rotating magnetic fields to induce magnetically steered locomotion of the microrobots (Movie S2). Controlled actuation of the microrobots was investigated in the range of magnetic frequencies of 1–30 Hz at a consistent magnetic field of 5 mT. Figure 3f clearly shows that the rate of microrobots’ displacement can be controlled using a desired frequency. As depicted in Figure 3g, the average speed of the microrobots was also dependent on the applied frequency. Initially, the actuation of the microrobots increased until the frequency of 5 Hz (step-out frequency), and then, it dropped gradually. At the step-out frequency, the microrobots reached their maximum average speed of ≈10 μm s^–1^.

### 2.3 Microrobots’ Selective Detection of Picric Acid

**Figure 4:**
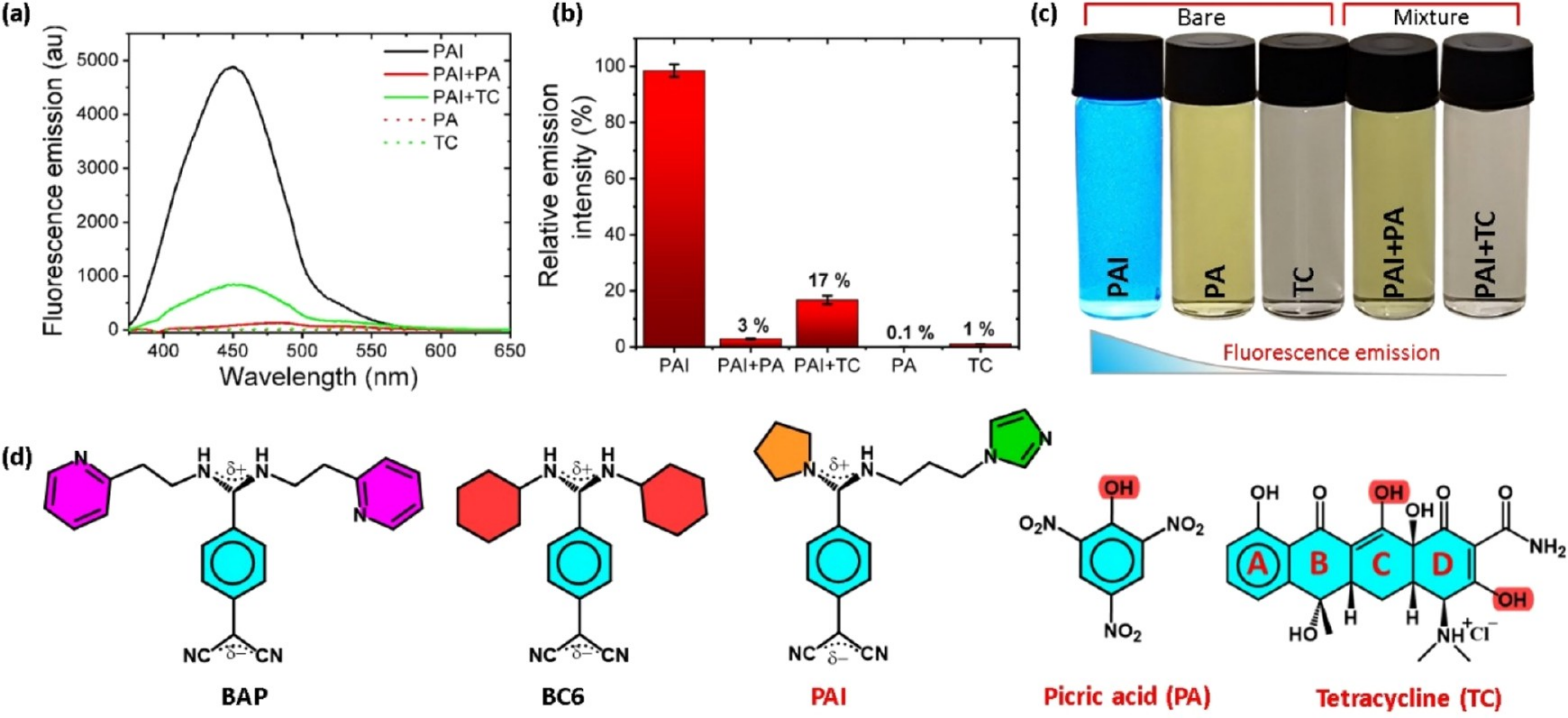
Microrobots sensing. (a) Emission spectra (*E*_x_: 350 nm), (b) relative fluorescence intensity variation, and (c) photographs of the vials filled with PAI microrobots before and after picric acid (PA) and tetracycline (TC) exposure. All photographs were captured under UV-light illumination. (d) Molecular structures of reference fluorescent materials (BAP and BC6), microrobots (PAI), picric acid, and tetracycline used in this study.

**Figure 5:**
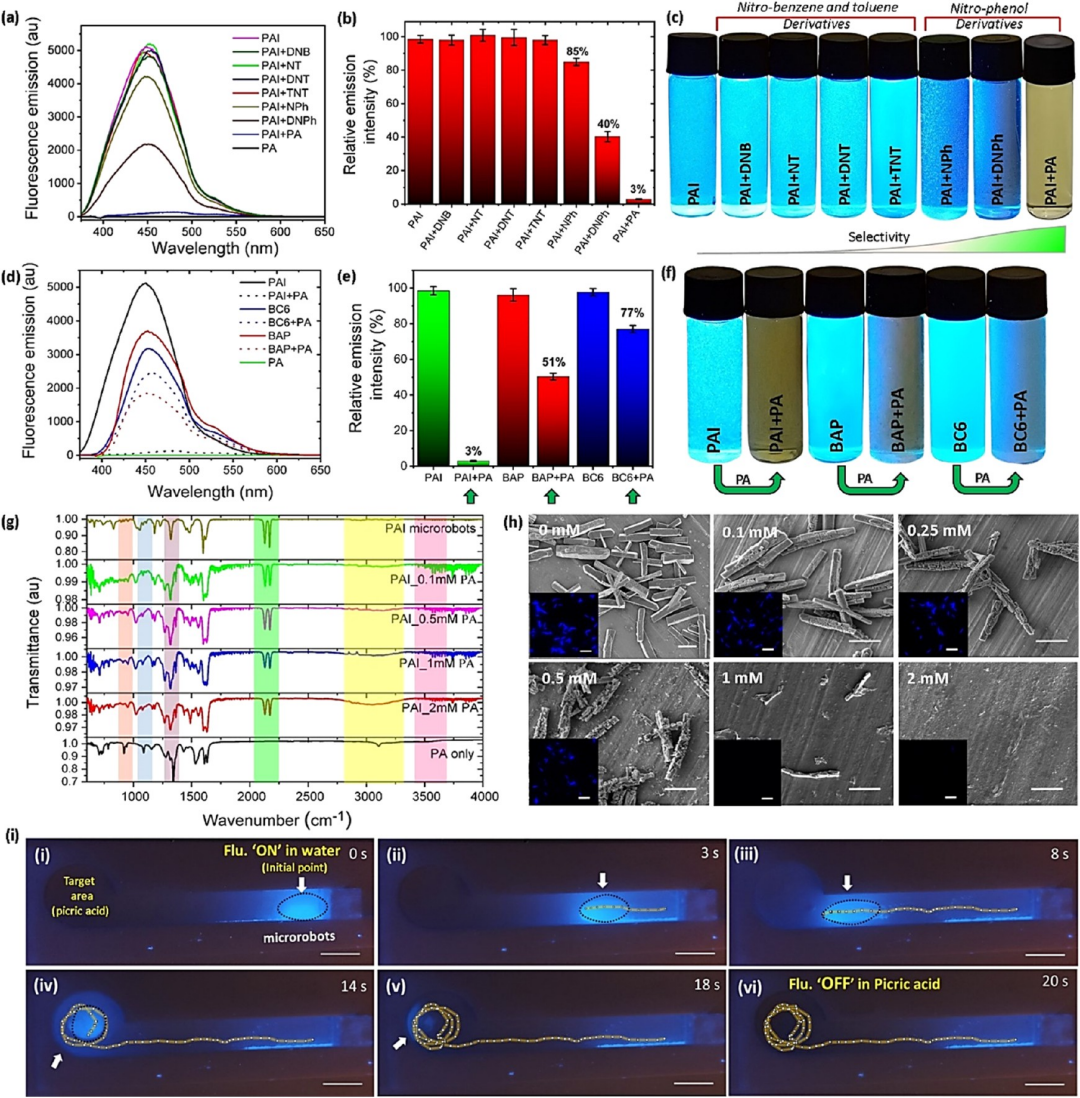
Microrobots’ selective detection of picric acid. (a–d) Fluorescence emission spectra (*E*_x_: 350 nm) and (b–e) relative fluorescence intensity variation. (c) Photographs of vials filled with PAI microrobots before and after exposure to various nitroaromatic compounds and (f) photographs of vials filled with PAI microrobots and BAP and BC6 compounds before and after exposure to picric acid. (g) FTIR spectra and (h) FESEM images of PAI microrobots with increasing concentrations of picric acid (scale bars: 10 μm). Insets show the corresponding fluorescence microscopy images (scale bars: 50 μm). (i) Time-lapse images of the externally guided locomotion of microrobots to the target area filled with picric acid in the fluidic channel (scale bars: 1 cm). All of the photographs of microrobots in vials and fluidic channel were captured under UV-light illumination.

The building blocks of the microrobots are engineered with amino groups capable of binding to target molecules for sensing applications. In this study, we have chosen nitroaromatic explosives (picric acid) and antibiotics (tetracycline) as target molecules to be detected employing magneto-fluorescent microrobots. The fluorescence response of the microrobots in the presence of target molecules is summarized in Figure 4a–4c. The result indicates that microrobots lost 97 and 83% of their fluorescence after treating with picric acid and tetracycline, respectively, as shown in Figure 4a,b. Notably, tetracycline has about 1% autofluorescence, which also contributes to the 17% fluorescence of PAI microrobots in a solution of tetracycline. Photographic images of the vials filled with microrobots before and after treatment with target materials under UV-light illumination are collected in Figure 4c, and the results are in agreement with our earlier observations. The molecular structure of the primary chemical compounds employed in this study, including PAI microrobots, reference fluorescent materials (BC6 and BAP, synthesis, and their NMR spectra, are shown in Figures S7 and S8), and target molecules, i.e., picric acid and tetracycline, are shown in Figure 4d. Electronic absorption and fluorescence emission spectra of fixed concentrations of PAI microrobots with increasing concentrations of picric acid are collected in Figure S9a,b, respectively. The results clearly show that the fluorescence emission of the microrobots gradually quenches with increasing concentrations of picric acid while absorption gradually increases, confirming the sensing potential of PAI microrobots toward picric acid. Then, PAI microrobots were treated with several other nitroaromatic explosives, including 1,3-dinitrobenzene (DNB), 4-nitrotoluene (NT), 2,6-dinitrotoluene (DNT), 2,4,6-trinitrotoluene (TNT), 4-nitrophenol (NPh), 2,4-dinitrophenol (DNPh), and 2,4,6-trinitrophenol (picric acid, PA), and their fluorescence emission response was recorded to confirm the selectivity of microrobots toward picric acid (Figure 5a–5c). The molecular structures of all nitroaromatic explosives employed in this work are summarized in Figure S10. The emission spectra comparison (Figure 5b) clearly reveals that PAI microrobots experience fluorescence loss exclusively with the nitrophenol derivatives but, interestingly, not with other nitroaromatic compounds. Moreover, among the nitrophenol derivatives, mononitro-substituted phenol (nitrophenol) decreases the fluorescence of the PAI microrobots by up to 15%, while dinitro (dinitrophenol) and trinitro (picric acid)-substituted phenols decrease up to 60 and 97% of the PAI microrobots’ fluorescence, respectively. Hence, nitro functionalities in picric acid offer selectivity for PAI microrobots. In addition, even though PAI microrobots exhibit its selectivity toward all three nitrophenol derivatives, namely, nitrophenol, dinitrophenol, and trinitrophenol (picric acid), the detection efficiency of PAI microrobots toward nitrophenol (15%) and dinitrophenol (60%) are poor compared to trinitrophenol (97%) and also not enough to be detected in real aqueous environments. Thereby, PAI microrobots could not be employed for their detection in real-world scenarios. Photographs of the vials having the solution of PAI microrobots with the other nitro compounds further confirm the selectivity of PAI microrobots toward picric acid (Figure 5c). In order to comprehend the role of functionalities in PAI molecules that enable sensing capability and selectivity, we employed two fluorescent compounds with similar molecular structures of PAI molecules as reference controls rather than PAI microrobots in the sensing experiments. The first one is 2-(2-aminoethyl)pyridine based DADQ derivative (7,7-bis(2-(2-aminoethyl)pyridino)–8,8-dicyanoquinodimethane, BAP) with less basic nitrogen functionalities,^56^ and the second one is a cyclohexylamine-based DADQ derivative (7,7-bis(cyclohexylamino)–8,8-dicyanoquinodimethane, BC6) with no nitrogen functionalities. According to their fluorescence analysis (Figure 5d,5e), picric acid reduces only 23% of BC6 molecules’ fluorescence, whereas it decreases the fluorescence of BAP and PAI compounds by up to 49 and 97%, respectively. In addition, photographs of the vials having solutions of PAI microrobots and BAP and BC6 compounds with picric acid confirm that imidazole functionalities in PAI molecules are underlying their selective sensing of picric acid (Figure 5f). Through these fluorescence analyses, we can hypothesize that interactions between the imidazole functionalities of PAI microrobots and the hydroxyl groups of picric acid molecules are critical in the sensing mechanism (Figure S11).

In order to gain deep insights into the picric acid sensing mechanism, FTIR, FESEM, and fluorescence microscopic analyses were carried out (Figure 5g,h). For these investigations, PAI microrobots were exposed to increasing concentrations of picric acid, as mentioned in the experimental section. The main motive of the FTIR experiment in this study is to establish a correlation between the fluorescence quenching of PAI microrobots and hydrogen binding interactions between the PAI and picric acid/tetracycline molecules while increasing the concentration of picric acid/tetracycline. The analysis clearly demonstrated that the fluorescence quenching increases as hydrogen bonding interactions intensify with increasing concentrations of picric acid/tetracycline. In order to prove the sensing mechanism without any ambiguity, we specifically analyzed only the peaks of picric acid/tetracycline, which showed a decrease with increasing concentration of picric acid/tetracycline after the interactions with PAI molecules. By focusing only these diminishing peaks, we ensured that the discussed spectral changes are due to only the interactions between the fluorescent probes and target analytes and not influenced by increasing concentrations of analytes. The disappearance of the hydroxyl peak of picric acid at 3440 cm^–1^ and the appearance of a new broad peak at 3063 cm^–1^ after exposing PAI microrobots to picric acid suggest proton transfer between the hydroxyl group of picric acid and nitrogen in the imidazole functionalities of PAI through strong hydrogen bonding interactions, leading to the formation of new charge transfer picrate complexes.^62−64^ The peaks of C–O bending and NO_2_ symmetric stretching vibrations of picric acid at 1086 and 1339 cm^–1^, respectively, are diminished significantly after the interaction with PAI molecules. As discussed earlier, the proton is transferred from picric acid to PAI molecules through hydrogen bonding, which leads to the formation of charge transfer complexation. This causes delocalization of the electronic density on the picrate moiety, which affects the symmetric and asymmetric vibrations of the NO_2_ functionalities of picric acid. Similarly, the C–H out-of-plane bending vibrations of picric acid at 919 cm^–1^ are also diminished. In addition, the peak positions of the nitrile groups of PAI microrobots at 2131 and 2172 cm^–1^ remain unchanged with increasing concentrations of picric acid, suggesting that nitrile groups of PAI molecules in microrobots, a potential functionality for electrostatic interactions, are not involved in any interactions (Figure 5g), supporting our hypothesis on the picric acid sensing mechanism. In turn, the structural stability and photophysical properties of PAI microrobots in picric acid were studied. PAI microrobots’ surface is gradually eradicated with increasing concentrations of picric acid, which is clearly illustrated in FESEM images (Figure 5h). Similarly, the fluorescence of PAI microrobots disappears with increasing concentrations of picric acid, as shown in fluorescence microscopic images (insets in Figure 5h). By correlating the results from all of the above experiments, the sensing mechanism was elucidated. Upon encounter with PAI microrods, the picric acid donates a proton from its acidic hydroxyl groups to the basic imidazole groups of PAI microrobots through strong hydrogen bonding, leading to the formation of new charge transfer picrate complexes that dissolve in water. The formation of CT complexes on the surface of the microrobots increases with increasing concentrations of picric acid (Figure 5g), which increases the structural degradation and fluorescence quenching of the microrobots, and eventually, it dissolves completely in water, as shown in Figure 5h. Previously, DPZDQ derivative, 7,7-bis(piperazine)-8,8-dicyanoquinodimethane, was reported as a nontoxic compound.^65^ Considering similarities between molecular frameworks of PAI and DPZDQ, even if PAI microrobots dissolve in the processes of picric acid detection, they are expected to have a limited effect in the surrounding area.

To validate the proposed sensing mechanism, FTIR analysis was carried out on BC6 molecules with increasing concentrations of picric acid (Figure S12). The results reveal that no new strong peaks formed around 3000 cm^–1^, while the hydroxyl peak of picric acid at 3440 cm^–1^ has disappeared.^62−64^ In addition, the peak positions of the nitrile groups of BC6 at 2132 and 2178 cm^–1^ are not changed even after exposure to picric acid. All of these observations suggest that only weak interactions are possible between the hydroxyl group of picric acid and the NH group in the diaminomethylene moiety of BC6. These weak interactions are likely to reduce the fluorescence of BC6 by only 23% (Figure 5e) upon interaction with picric acid. Then, the limit of detection (LOD) of picric acid was calculated using the formula 3.3σ/*k* (Figure S13), where *k* defines the slope from the calibration curve of graph plotting between the increasing concentration of the picric acid and fluorescence intensity of PAI molecules and σ represents the standard deviation of the fluorescence intensities of a series of blank PAI molecules before treating with picric acid. The LOD is found to be 214 nM.

To investigate the feasibility of practical implementations of microrobots for real-world sensing applications, a fluidic channel was employed as a testing platform, as illustrated in Figure 5i. In this experiment, the long-range magnetic maneuverability is demonstrated by directing the magneto-fluorescent microrobots toward a target area of the fluidic channel, which is several centimeters away from their starting point, under the influence of a magnetic field. Time-lapse images of the microrobots in the fluidic channel were recorded under UV-light illumination (Movie S3). Figure 5i(i) depicts the presence of PAI microrobots having blue fluorescence at the initial point of the fluidic channel where microrobots started their journey. Figure 5i(ii),(iii) illustrate the navigation of microrobots toward the target area filled with picric acid under the influence of a permanent magnet. After reaching the target area, microrobots were navigated in a circular pattern over several seconds, as depicted in Figure 5i(iv),(vi), which enhanced the interactions between PAI microrobots and picric acid. From Figure 5i(iv)–(vi), the fluorescence of microrobots was gradually diminished, and finally, the fluorescence was fully quenched due to extensive interactions between PAI microrobots and picric acid. For the control experiments, a similar procedure was repeated with dinitrotoluene in the target area of the fluidic channel (Figure S14). As expected, the fluorescence of microrobots did not quench throughout the entire experiment, indicating the absence of any interactions between dinitrotoluene and PAI microrobots in the target area. Lastly, analytical features of various fluorescent probes for picric acid detection are compared with PAI microrobots in Table S2.

### 2.4 Microrobots’ Detection of Tetracycline

**Figure 6:**
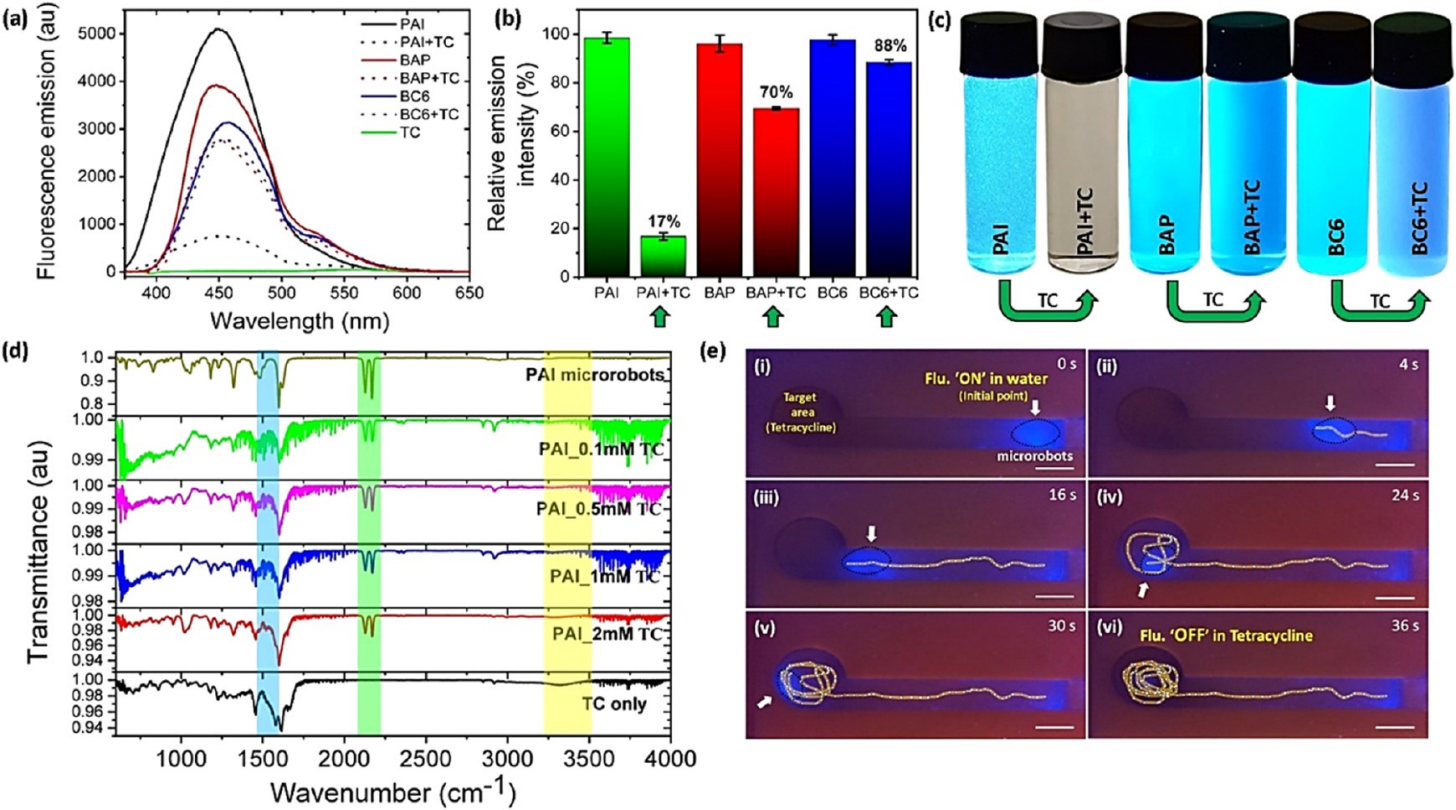
Microrobots’ detection of tetracycline. (a) Fluorescence emission spectra (*E*_x_: 350 nm), (b) relative fluorescence intensity comparison, and (c) photographs of vials filled with PAI microrobots, BAP, and BC6 before and after exposure to tetracycline. (d) FTIR spectra of PAI microrobots with increasing concentration of tetracycline (TC). (e) Time-lapse images of the externally guided locomotion of the microrobots toward a target area filled with tetracycline in the fluidic channel (scale bars: 1 cm). All photographs were captured under UV-light illumination.

The fluorescence “on–off” sensing strategy was tested once again for the detection of tetracycline. To this end, a fixed concentration of PAI microrobots was treated with increasing concentrations of tetracycline, and their photophysical properties were analyzed. The analyses show that fluorescence emission of PAI microrobots decreases with increasing concentration of tetracycline while absorption increases, as with picric acid sensing (Figure S15a,b). Like the previous case, PAI microrobots and fluorescent compounds (BAP and BC6) were treated with tetracycline, and their fluorescence variations were analyzed to elucidate the sensing mechanism (Figures 6a,6b). As expected, in the presence of tetracycline, PAI microrobots lost 83% fluorescence, whereas BAP and BC6 lost only 30 and 12%, respectively. This observation was once again confirmed by photographs of the glass vials filled with PAI microrobots and BAP and BC6 compounds in tetracycline (Figure 6c). This significant loss observed in PAI microrobots’ fluorescence is due to their imidazole functionality, which is not present in BC6 and BAP compounds. Unlike previous cases, BC6 lost only 12% of its fluorescence upon the interactions with tetracycline, which demonstrates the absence of any significant strong interactions. Steric hindrance, resulting from the bulky molecular structure of tetracycline and the limited space around diaminomethylene moiety of BC6 compounds, prevents the efficient interactions between the tetracycline’s hydroxyl group and amine group in the diaminomethylene moiety of BC6 compounds.

To confirm the role of the imidazole functionality of PAI microrobots in tetracycline sensing, FTIR analysis was carried out on PAI microrobots with increasing concentrations of tetracycline (Figure 6d). The peaks of C = O stretching vibrations of the D and B benzene rings of tetracycline at 1576 and 1616 cm^–1^, respectively, and the peak of amide (C=O group) functionality of tetracycline at 1661 cm^–1^ decrease with increasing concentration of tetracycline confirms the interactions between the tetracycline and PAI microrobots. Moreover, decreasing OH stretching vibrations of tetracycline at 3336 cm^–1^ reflect the proton transfer interactions between the hydroxyl group of tetracycline and the imidazole functionality of PAI microrobots. Unlike picric acid sensing, no new peak was observed around 3000 cm^–1^ in the mixture of PAI microrobots and tetracycline, indicating the absence of hydrogen bonding interactions between them.^66^ According to existing DFT calculations in the literature survey, the hydroxyl group in the phenyl ring D is more acidic than the other hydroxyl groups of tetracycline and experiences less steric hindrance.^67^ Consequently, during interactions with PAI microrobots, tetracycline most likely transfers its proton from the hydroxyl group of phenyl ring D to the imidazole of PAI microrobots, and their plausible mechanism is shown in Figure S16. Additionally, a second proton transfer from hydroxyl functionalities present in the phenyl ring C is also feasible.^68^ Then, the structural integrity and photophysical properties of the microrobots were analyzed at different concentrations of tetracycline (Figure S17). Like picric acid sensing, tetracycline gradually degrades the surface of the microrobots, leading to complete dissolution at higher concentrations of tetracycline, as shown in Figure S17a. Fluorescence microscopy analysis also endorses the gradual degradation of microrobots (Figure S17b). The LOD of tetracycline is found to be 350 nM for the PAI molecules (Figure S18). According to the fluorescence emission analysis and LOD calculations, it is clear that PAI microrobots are more efficient for the detection of picric acid than tetracycline. At last, the fluidic channel was employed to investigate the potential for long-range operation of PAI microrobots for tetracycline sensing (Movie S4). A few drops of microrobots were placed at the initial point of the fluidic channel as shown in Figure 6e(i) and then the microrobots were magnetically directed toward the target area filled with tetracycline (Figure 6e(ii),(iii)). After reaching the target area, the microrobots were circularly manipulated for several minutes, and their fluorescence was monitored under the illumination of UV-light. As expected, the microrobots gradually lost their fluorescence with time until its complete disappearance (Figure 6e(iv)–(vi)). Overall, the fluidic channel-associated experiments emphasize the potential of magneto-fluorescent microrobots for remote sensing applications. In addition, analytical features of PAI microrobots for tetracycline detection are compared with various reported fluorescent probes in Table S3.

Since we are interested in monitoring pollutants in real aqueous environments, it is critical to study the impact of potential factors like pH and ionic species on the detection performance of PAI microrobots. For this reason, we studied the fluorescence response of PAI microrobots to picric acid/tetracycline in the Britton–Robinson (BR) buffer solutions with pH range from 2 to 11 (Figure S19) as well as in the presence of various ionic species (Figure S20). By comparing the fluorescing quenching of PAI molecules by picric acid/tetracycline in neutral (pH 7), basic (pH 8–11), and acetic (pH 2–5) conditions, it clearly shows that fluorescence of PAI microrobots remains unchanged before and after being treated with picric acid/tetracycline under acidic conditions. On the other hand, in basic conditions, PAI microrobots exhibit higher fluorescence than after being treated with picric acid/tetracycline. The results illustrate that the fluorescence quenching caused by an acidic medium is much stronger than that caused by picric acid/tetracycline due to the strong protonation of imidazole functionalities of PAI molecules in acidic medium, which is absent in neutral and basic mediums. Hence, the picric acid/tetracycline detection performance of PAI microrobots is strongly influenced in acidic medium (pH 2–5) but not in neutral and basic pH medium (pH 6–11). Overall, Figure S19 clearly highlights the efficacy of PAI microrobots for sensing applications in neutral and basic conditions. Lastly, the effect of various ionic species on the detection performance of PAI microrobots for picric acid was also studied. Figure S20 clearly illustrates that Ca^2+^, CO^2+^, Cu^2+^, Fe^2+^, K^+^, Na^+^, Ni^2+^, and Zi^2+^ ions do not have any significant effect on the picric acid detection performance of PAI microrobots.

## 3 Conclusions

In this study, we developed molecularly engineered magneto-fluorescent microrobots functionalized with imidazole groups having a decoration of Fe_3_O_4_ nanoparticles for the selective detection of picric acid in the presence of various nitroaromatic compounds and tetracycline. The microrobots displayed collectability and controllable wireless actuation capabilities in the presence of magnetic fields. Fluorescent microrobots were shown to exhibit fluorescence quenching upon exposure to either picric acid or tetracycline, and we have highlighted their efficient utilization for the detection of these target molecules in real-world scenarios using a fluidic channel as a testing platform. Furthermore, fluorescence switching of PAI microrobots following substantial locomotion toward target nitroaromatic compounds in the fluidic channel validated their intrinsic magnetic maneuverability and selective sensitivity. Extensive spectroscopic and microscopic analyses provide useful insights into the molecular-level changes in the microrobots, including the formation of charge transfer complexes that cause fluorescence quenching of PAI microrobots upon exposure to either picric acid or tetracycline. This present study opens new avenues for designing a new class of magneto-fluorescent molecular material-based microrobots, which can be employed for various applications, including homeland security, forensic analysis, and industrial safety.

## 4 Experimental Section

### 4.1 Materials

7,7,8,8-tetracyanoquinodimethane (TCNQ, C_12_H_4_N_4_, 98%), pyrrolidine (C_4_H_9_N, 99%), 1-(3-aminopropyl)imidazole (C_6_H_11_N_3_, ≥97%), cyclohexylamine (C_6_H_11_NH_2_, ≥99%), 2-(2-aminoethyl)pyridine (C_7_H_10_N_2_, 95%), iron(ii)sulfate heptahydrate (FeSO_4_·7H_2_O, ≥99%), ferric chloride hexahydrate (FeCl_3_·6H_2_O, ≥98%), calcium chloride dihydrate (CaCl_2_·2H_2_O, ≥99%), picric acid (PA, 1.3% in H_2_O), 1,3-dinitrobenzene (DNB, 97%), 4-nitrotoluene (NT, 99%), 2,6-dinitrotoluene (DNT, 98%), 2,4,6-trinitrotoluene (TNT, 10 mg/mL in acetonitrile solution), 4-nitrophenol (NPh, 99%), 2,4-dinitrophenol (DNPh, 98%) were purchased from Sigma-Aldrich and used for the experiments without any further purification. In addition, HPLC grade solvents were used for the synthesis of PAI, BAP, and BC6. Ultrapure water was used in the synthesis of Fe_3_O_4_ nanoparticles, fabrication of PAI microrobots, and preparation of dilute solution of nitroaromatic compounds. TNT was received from Sigma-Aldrich in an acetonitrile solution; prior to use, it was dried and dissolved in water.

### 4.2 Synthesis of PAI Molecules

The two-step synthesis of the PAI compound is shown in Figure 1a. In the first step of synthesis of 7-pyrrolidino-7,8,8-tricyanoquinodimethane (PTCNQ), pyrrolidine (1.22 mmol, 87 mg, 102 μL) was added to a stirred solution of TCNQ (1.22 mmol, 250 mg) in 20 mL of acetonitrile at 70 °C and then further stirred for 3 h. The yellow solution of TCNQ turned purple immediately after the addition of pyrrolidine. Purple precipitate was filtered, washed, and dried (yield 88%). PTCNQ was recrystallized from its acetonitrile solution. ^1^H NMR (60 MHz, DMSO-*d*_6_) δ 7.68 (d, *J* = 9 Hz, 2H), 6.91 (d, *J* = 8.4 Hz, 2H), 4.13 (M, 4H), δ 2.05 (M, 4H). In the second step, 1-(3-aminopropyl)imidazole (1.3 mmol, 165 mg, 158 μL) was added to a solution of PTCNQ (0.6 mmol, 150 mg) in acetonitrile (15 mL) at 70 °C for 3 h. The purple solution of PTCNQ immediately turned to yellow upon the addition of amines. The resulting white precipitate was filtered, washed with acetonitrile, and dried (yield 90%). ^1^H NMR (60 MHz, DMSO-*d*_6_) δ 8.32 (s, 1 H), 7.39 (s, 1 H), 6.90 (d, *J* = 8.4 Hz, 2H), 6.70 (d, *J* = 5.4 Hz, 4H), 3.66 (t, *J* = 6.6 Hz, 2H), 3.05 (m, 6H), 1.75 (m, 6H). MALDI-MS: M calc. for C_20_N_6_H_22_ = 346.43 Da; found: 347.198 Da [M + H]^+^. PAI compounds were recrystallized using acetonitrile, and the resulting crystals were used for SCXRD analysis and fabrication of microrobots. For the synthesis of BAP and BC6, 2-(2-aminoethyl)pyridine (1.22 mmol, 150 mg, 146 μL) or cyclohexylamine (1.96 mmol, 194 mg, 225 μL) was separately added into a stirred solution of TCNQ (0.49 mmol, 100 mg) in acetonitrile (10 mL) at 72 °C for 3 h, respectively.

### 4.3 Preparation of Fe_3_O_4_ Nanoparticles

At 25 °C, 20 mg of CaCl_2_·2H_2_O and 38 mg of FeSO_4_·7H_2_O were each dissolved in 20 mL of water. Then, this solution was thoroughly mixed with 37 mg of FeCl_3_·6H_2_O at 60 °C for 15 min. To this solution, 27% of ammonium hydroxide was added dropwise until the pH of the solution reached 11; finally, the solution was further stirred for 15 min. The resulting Fe_3_O_4_ nanoparticles were collected using a permanent NdFeB magnet and washed several times with water and ethanol. The collected Fe_3_O_4_ nanoparticles were dried overnight at room temperature.

### 4.4 Fabrication of Microrobots

0.5 mL DMSO solution of PAI (4 mg) was injected into 5 mL of water under sonication for 30 s to fabricate PAI nanocrystals. Then, 1 mL of aqueous solution of Fe_3_O_4_ nanoparticles (1 mg in 10 mL of water) was injected into the solution of nanosized PAI crystals and left undisturbed for 1 h. In this aging time of 1 h, the nanosized crystals grew to microsized crystals while Fe_3_O_4_ nanoparticles were attached to their surface. The resulting PAI microcrystals with a decoration of Fe_3_O_4_ nanoparticles were collected by using a permanent magnet and then employed for actuation studies and sensing applications.

### 4.5 Spectroscopic Analyses

A Jasco V-750 UV–vis spectrophotometer and a Jasco FP8300 spectrofluorometer were employed for recording the electronic absorption and fluorescence emission spectra, respectively. All emission spectra of PAI solution, microcrystalline solids, microrods, and microrobots were recorded by exciting at 350 nm. Acetonitrile solution of PAI (0.1 mM) and the PAI microcrystalline solids were employed to record their absorption and emission spectra. For the picric acid/tetracycline sensing experiment, 0.5 mL of PAI microrobots and 0.5 mL of different concentrations (0.05 0.1, 0.25, 0.5, 1, 1.5, and 2 mM) of either picric acid or tetracycline were mixed with 3.5 mL of water, and their fluorescence emission spectra were recorded. In the experiments of establishing the selective picric acid sensing of PAI microrobots, fluorescence emission spectra were recorded for the solution mixtures of 0.5 mL of PAI microrobots, 0.5 mL of any nitroaromatic explosives (2 mM), including 1,3-dinitrobenzene, 4-nitrotoluene, 2,6-dinitrotoluene, 2,4,6-trinitrotoluene, 4-nitrophenol, 2,4-dinitrophenol, 2,4,6-trinitrotoluene (picric acid), and 3.5 mL of water. In the experiment to establish the role of functionalities in PAI microrobots for sensing, fluorescence emission spectra were recorded for the solution mixture of 0.5 mL of PAI microrobots/BAP/BC6, 0.5 mL of a 2 mM solution of target molecules (picric acid or tetracycline), and 3.5 mL of water. For the LOD analysis, a stock solution was prepared by dissolving 4 mg of PAI in 3 mL of DMSO. Then, 50 μL of stock solution was mixed thoroughly with 2 mL of different concentrations (0.1, 0.5, 2, 4, 6, 8, and 10 μM) of either picric acid or tetracycline for their fluorescence emission spectra. A Vertex 70v FTIR spectrometer coupled with a Hyperion 3000 microscope was employed for FTIR analysis. For FTIR spectral analysis, 0.5 mL of picric acid/tetracycline with different concentrations was mixed with 0.5 mL of PAI microrobots. To investigate the effect of pH, 0.5 mL of PAI microrobots and 0.5 mL of 1 mM picric acid/tetracycline were mixed with 3 mL of Britton–Robinson (BR) buffer solutions varying pH from 2 to 11 and their fluorescence emission spectra were recorded. To investigate the effect of ionic species, 0.5 mL of PAI microrobots, 0.5 mL of 1 mM picric acid/tetracycline, and 0.5 mL of 1 mM solution of ionic solutions were mixed with 3 mL of distilled water, and their fluorescence emission spectra were recorded. The mass spectrum was recorded on an UltrafleXtreme (Bruker Daltonics) mass spectrometer by reflection positive ion detection mode with α-cyano-4-hydroxycinnamic acid as a MALDI matrix.

### 4.6 Magnetic Actuation

A customized magnetic setup containing three orthogonal coil pairs housed in a poly(lactic acid)-based three-dimensional (3D)-printed holder coupled with a navigation controller was employed to actuate the microrobots. A Nikon Ts2R inverted microscope coupled with a Basler acA1920–155uc camera was employed to record the locomotion of the microrobots. Actuation of the microrobots was analyzed in a frequency range of 1 to 30 Hz at a constant magnetic field of 5 mT. Recorded movies were analyzed using NIS-Elements Advanced Research and FIJI software. Sodium dodecyl sulfate (0.15 wt %) was utilized for all motion experiments. The average speed of the microrobots under different conditions was estimated by tracking multiple microrobots.

### 4.7 X-ray Diffraction Analyses

Rigaku Oxford XtaLAB Synergy R custom diffractometer coupled with an automated crystal transport orientation and retrieval robot (ACTOR) was employed for the single-crystal X-ray diffraction analysis of a single crystal of PAI. Cu Kα radiation was generated with the assistance of a rotating anode X-ray tube, and data were collected using a hybrid pixel array detector at 120 K. CrysAlisPro version 1.171.42.95a and SHELXL-2019/3 software were used for reducing and refining the data, respectively. The powder X-ray diffraction spectra of PAI microcrystals were recorded employing a Rigaku Smart Lab 3 kW XRD diffractometer at 40 kV and 30 mA. Mercury 3.8 software was employed to derive the simulated powder X-ray diffraction pattern of the PAI crystal from its SCXRD data.

### 4.8 Microscopic Analyses

FEI Verios 460L FESEM and Tescan MIRA3 XMU SEM coupled with an EDAX detector were employed to record the morphological features and elemental analysis of the PAI microrobots, respectively. The microrobots were drop-cast on copper tape, followed by a thin gold layer coating (10 nm) by a Leica EM ACE600 high-vacuum sputter-coater for the FESEM analysis. The fluorescence micrographs were recorded using a Nikon Ts2R inverted optical microscope equipped with a UV LED (CoolLED pE-100, 365 nm) and a digital camera (Basler, model acA1920–155uc). 40× and 60× objectives and DAPI filter (*E*_x_: 365 nm and *E*_m_: 445 nm) were used to record the fluorescence micrograph.

### 4.9 Fluid Channel-Associated Experiments

A Teflon-based fluid channel was employed for the real-time sensing experiments. Prior to the experiment, the channel was filled with 5 mL of water. Then, a few drops of PAI microrobots were added to the initial point, while 1 mL of 4 mM solution of picric acid/tetracycline/dinitrotoluene was added to the target area of the fluidic channel, and the microrobots were navigated toward the target area with the help of a permanent magnet. The fluorescence response of the microrobots was monitored and recorded under UV-light illumination.

## Supporting Information Available

Structural characterization of PAI molecules: NMR and mass spectroscopic, and single crystal X-ray diffraction analyses; schematic representation for the fabrication of PAI microrobots; PXRD and VSM analysis of Fe3O4 nanoparticles; synthesis and NMR spectroscopic analysis of BAP and BC6; fluorescence detection of picric acid/tetracycline using PAI microrobots and their plausible reaction mechanism; limit of detection of picric acid/tetracycline for PAI microrobots; FTIR spectra of BC6 with picric acid and tetracycline; locomotion of PAI microrobots towards dinitrotoluene (DNT) in the testing platform; effect of picric acid/tetracycline on morphology and fluorescence of PAI microrobots; effect of pH and metal ionic species on the detection performance of PAI microrobots for both picric acid and tetracycline; comparison tables of detection performance of PAI microrobots with that of other fluorescent sensing probes to both picric acid and tetracycline (PDF)
Motion of PAI microrobots in a fluidic channel filled with water under the influence of a magnet (Movie S1) (MP4)
Motion of PAI microrobots under the influence of transversal rotating magnetic field (5 mT, 5 Hz) (Movie S2) (MP4)
Motion of PAI microrobots toward picric acid (PA, explosive) in the fluidic channel under the influence of a magnet (Movie S3) (MP4)
Motion of PAI microrobots toward tetracycline (TC, antibiotic) in the fluidic channel under the influence of a magnet (MP4)
