# Supplementary material for: Magneto-Fluorescent Microrobots with Selective Detection Intelligence for High-Energy Explosives and Antibiotics in Aqueous Environments
Source: ACS Appl Mater Interfaces. 2025 Mar 27;17(14):21691–704. doi: 10.1021/acsami.5c02259 (PMC11986900; doi:10.1021/acsami.5c02259)
Supplement: Supplementary file 1 — am5c02259_si_001.pdf [file am5c02259_si_001.pdf]

Magneto-Fluorescent Microrobots with Selective Detection Intelligence for High-energy Explosives and Antibiotics in Aqueous Environments

N. Senthilnathan[1], Cagatay M. Oral[1], Martin Pumera[1,2,3]

[1]Future Energy and Innovation Laboratory, Central European Institute of Technology, Brno University of Technology, Purkynova 123, Brno 61200, Czech Republic

[2]Advanced Nanorobots & Multiscale Robotics Laboratory, Faculty of Electrical Engineering and Computer Science, VSB - Technical University of Ostrava, 17. listopadu 2172/15, 70800 Ostrava, Czech Republic

[3]Department of Medical Research, China Medical University Hospital, China Medical University, No. 91 Hsueh-Shih Road, Taichung, Taiwan.

✉ email: martin.pumera@ceitec.vutbr.cz

**Page**	**Content**
S3	NMR spectroscopic characterization of PAI **(Figure S1)**
S4-S5	Single crystal X-ray diffraction analysis **(Figure S2, Table S1)**
S6	Mass spectroscopic analysis **(Figure S3)**
S7	Aggregation induced emission **(Figure S4)**
S8	Fabrication of PAI microrobots **(Figure S5)**
S9	PXRD and VSM analysis of Fe_3O_4 nanoparticles **(Figure S6)**
S10	Synthesis of BAP and BC6 **(Figure S7)**
S11	NMR spectroscopic characterization of BAP and BC6 **(Figure S8)**
S12	Detection of picric acid **(Figure S9)**
S13	Molecular structure of nitroaromatic explosives **(Figure S10)**
S14	Plausible reaction mechanism between PAI and picric acid **(Figure S11)**
S15	FTIR spectra of BC6 with picric acid and tetracycline **(Figure S12)**
S16	Limit of detection for Picric acid **(Figure S13)**
S17	Locomotion of microrobots towards dinitrotoluene (DNT) **(Figure S14)**
S18	Comparison of detection performance of PAI microrobots with other fluorescent sensing probes to picric acid **(Table S2)**
S19	Detection of Tetracycline **(Figure S15)**
S20	Plausible reaction mechanism between PAI and tetracycline **(Figure S16)**

S21 Effect of tetracycline on morphology and fluorescence of PAI microrobots **(Figure S17)**

S22 Limit of detection for Tetracycline **(Figure S18)**

S23 Comparison of detection performance of PAI microrobots with other fluorescent sensing probes to tetracycline **(Table S3)**

S24 Effect of pH on the detection performance of PAI microrobots to picric acid and tetracycline **(Figure S19)**

S25 Effect of metal ionic species on the detection performance of PAI microrobots to picric acid **(Figure S20)**

S26 References

NMR spectroscopic characterization of PAI

(a) 7-pyrrolidino-7,8,8-tricyanoquinodimethane (PTCNQ)

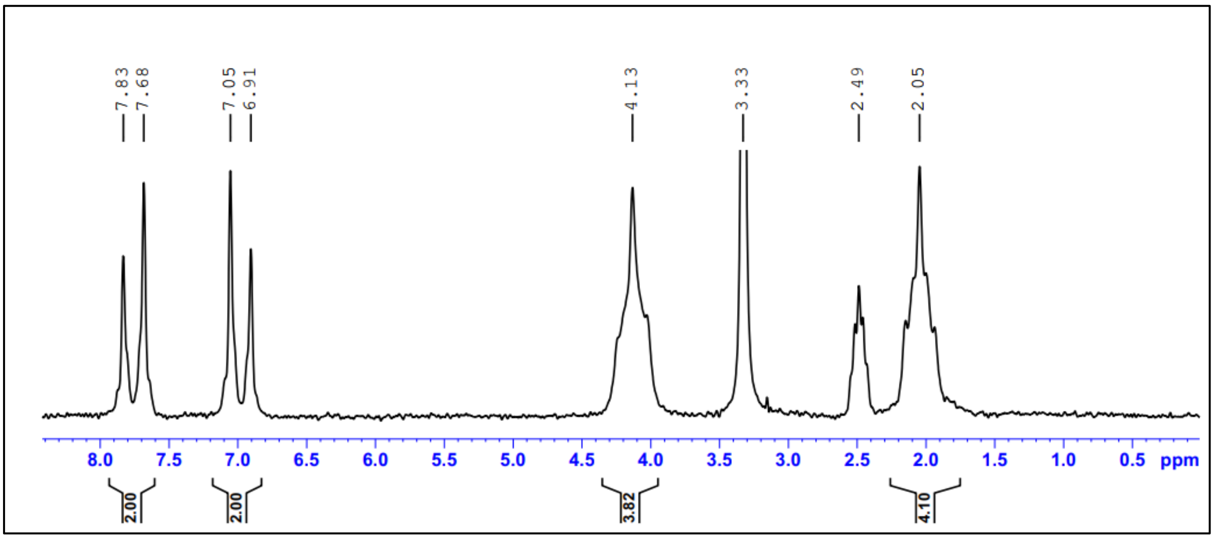

^{1}H NMR (60 MHz, DMSO-d_6) δ 7.68 (d, J = 9 Hz, 2H), δ 6.91 (d, J = 8.4 Hz, 2H), δ 4.13 (M, 4H), δ 2.05 (M, 4H).

(b) 7-pyrrolidino-7-(1-(3-aminopropyl)imidazole)-8,8-dicyanoquinodimethane (PAI)

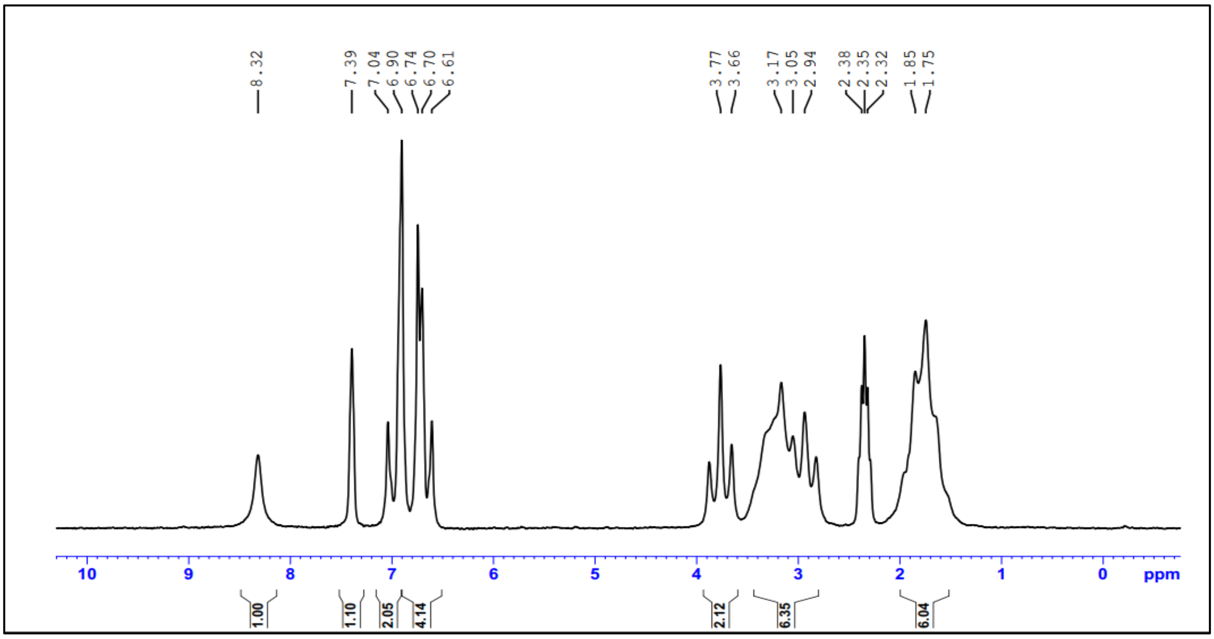

^{1}H NMR (60 MHz, DMSO-d_6) δ 8.32 (s, 1 H), δ 7.39 (s, 1 H), δ 6.90 (d, J= 8.4 Hz, 2H), δ 6.70 (d, J= 5.4 Hz, 4H), δ 3.66 (t, J= 6.6 Hz, 2H), δ 3.05 (m, 6H), δ 1.75 (m, 6H).

Figure S1. ^{1}H NMR spectra of (a) PTCNQ and (b) PAI.

Single crystal X-ray diffraction analysis

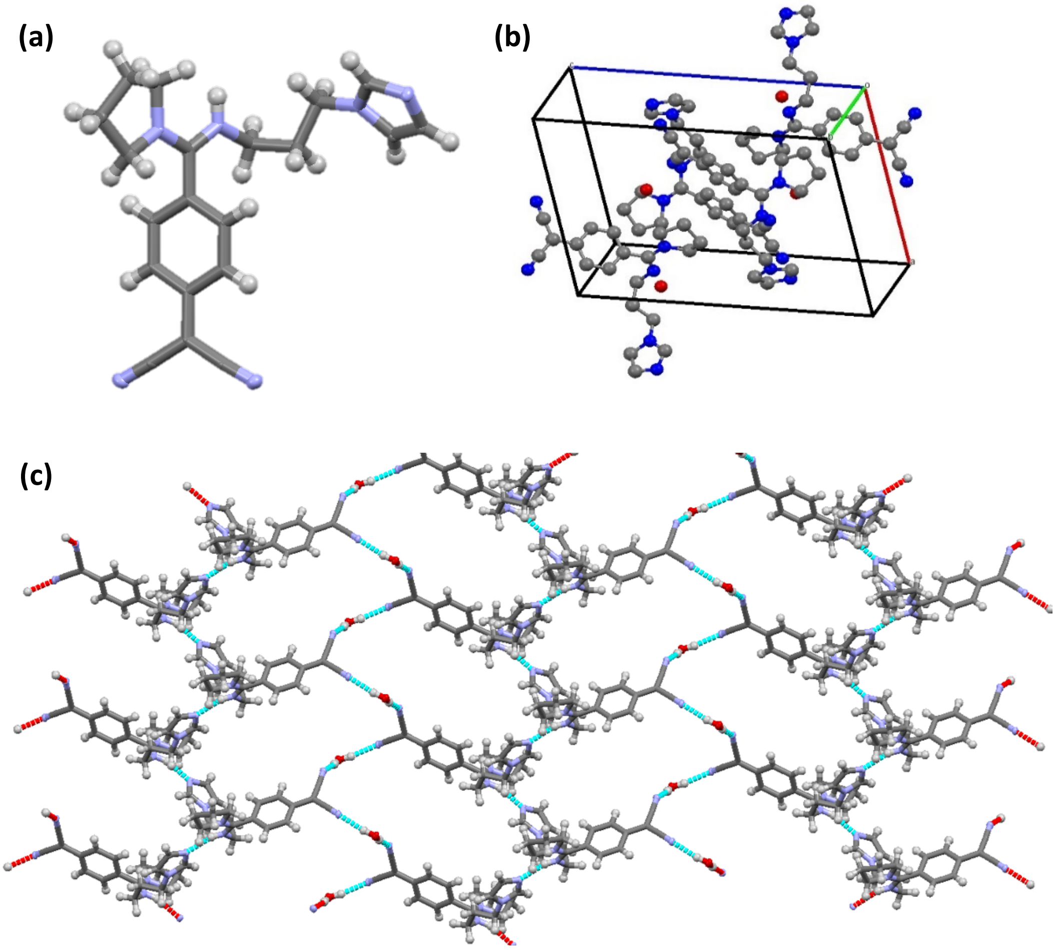

Figure S2. Single crystal X-ray diffraction analysis of PAI. (a) Molecular structure, (b) Unit cell packing, (c) Supramolecular assembly view along the b axis. Intermolecular hydrogen bonds are shown as cyan lines. Carbon, nitrogen, and oxygen atoms are displayed as grey, blue, and red balls, respectively, and hydrogen atoms are omitted for clarity.

Table S1. Basic crystallographic data of PAI

	**PAI**
Empirical formula	$C_{20}H_{24}N_6O$
Crystal system	monoclinic
Space group	$P2_1/n$
a / Å	11.87797(6)
b / Å	9.32603(4)
c / Å	17.39728(10)
α / deg.	90
β / deg.	102.4585 (5)
γ / deg.	90
V / Å^3	1881.792(17)
Z	4
$\rho_{calc.}$ / g cm^{-3}	1.286
μ / mm^{-1}	0.671
Temperature / K	120
λ / Å	1.54184
No. of reflections	3879
No. of parameters	250
Max., Min. transmission	0.874,0.904
GOF	1.058
R [for I ≥ $2\sigma_I$]	0.0344
wR^2	0.0859
Largest difference peak and hole / eÅ^{-3}	0.212/ -0.263
CCDC number	2404559

Mass spectroscopic analysis

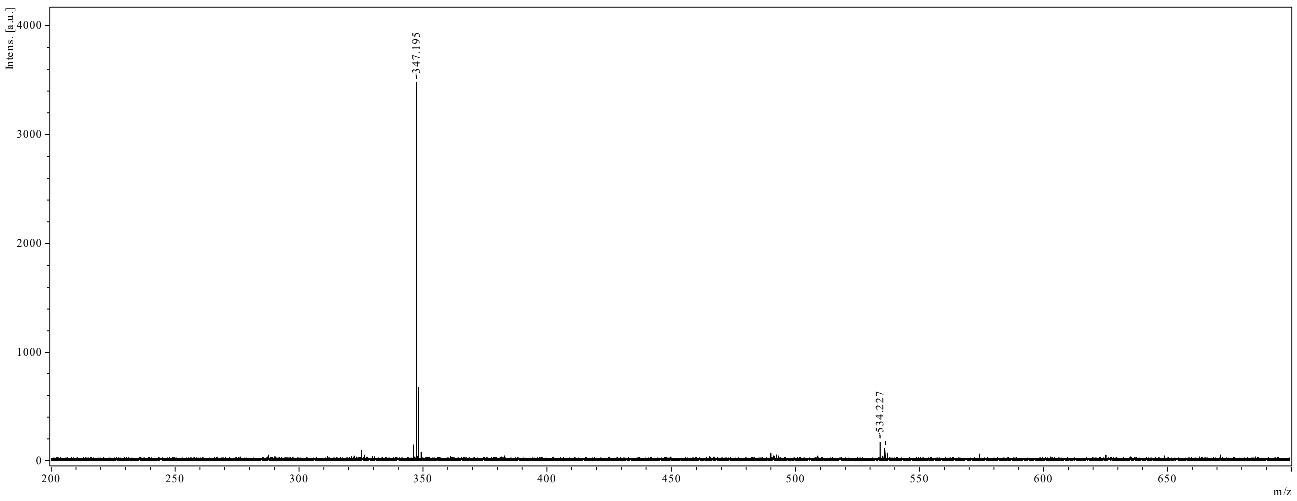

Figure S3. MALDI MS intact mass spectroscopic analysis of PAI.

The mass spectrum was recorded on an UltrafleXtreme (Bruker Daltonics) Mass Spectrometer by reflection positive ion detection mode with α-cyano-4-hydroxycinnamic acid as a MALDI matrix.

Two peaks were observed at 347 and 534 Da.

The theoretical calculation for M (PAI, $C_{20}N_6H_{22}$) is 346.43 Da; found: 347.198 Da $[M+H]^+$. The second peak at 534 Da is attributed to adduct of the PAI molecule and MALDI matrix, α-cyano-4-hydroxycinnamic acid.

Aggregation induced emission

100 µl of 0.1 µM solution of PAI (in DMSO) was added to the series of solvent mixtures containing water and DMSO with increasing water fraction from 0 to 0.95, and their fluorescence emission spectra were recorded.

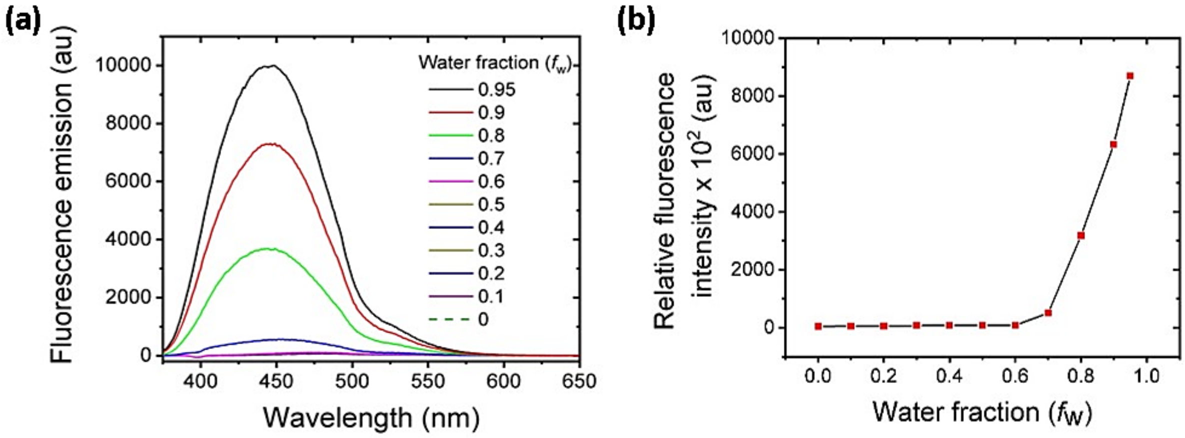

Figure S4. (a) Fluorescence emission spectra (λ_{exc}= 350 nm) and (b) corresponding fluorescence intensity variation of PAI in a solvent mixture containing DMSO and water with increasing water fraction from 0% to 95%.

Fabrication of PAI microrobots

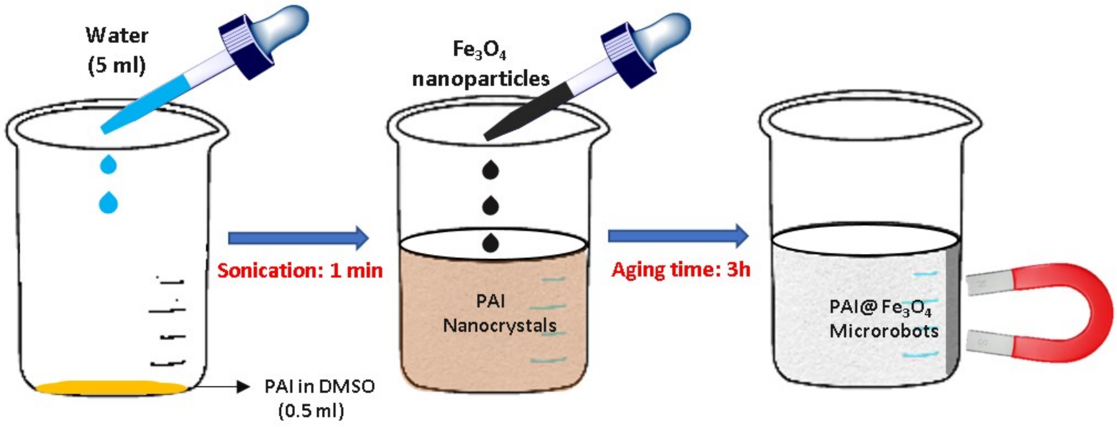

Figure S5. A pictorial illustration of the fabrication of PAI microrobots.

PXRD and VSM analysis of Fe_3O_4 nanoparticles

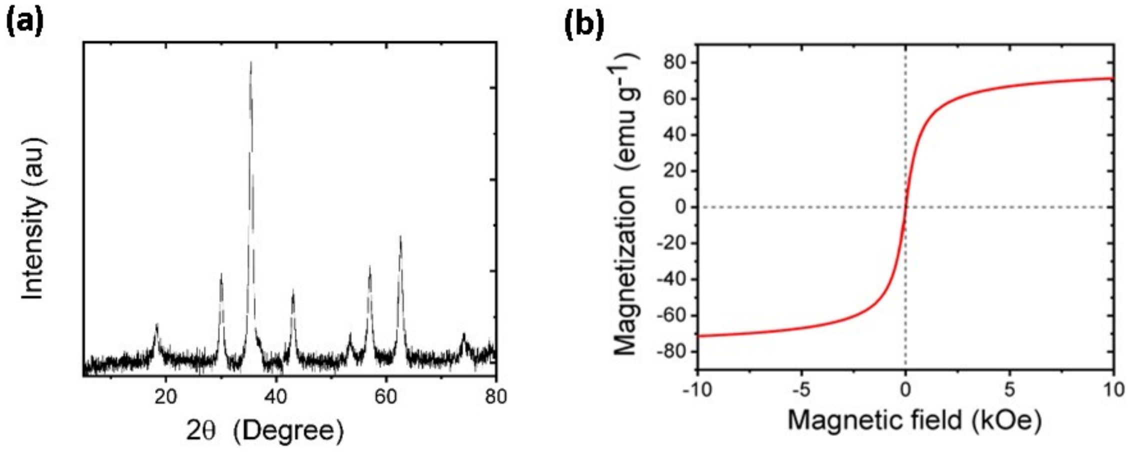

Figure S6. (a) PXRD pattern and (b) Magnetic hysteresis loop of Fe_3O_4 nanoparticles used for the fabrication of microrobots in this study.

Synthesis of BAP and BC6

(a) 7,7-bis(2-(2-aminoethyl)pyridino)-8,8-dicyanoquinodimethane (BAP)

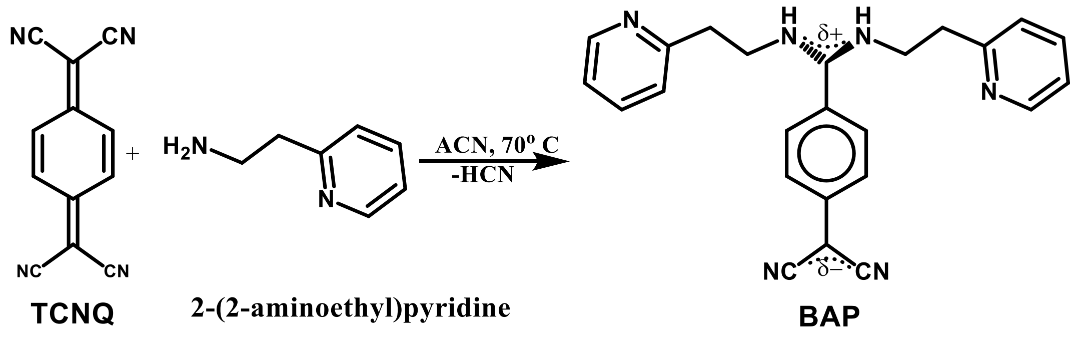

(b) 7,7-bis(cyclohexylamino)-8,8-dicyanoquinodimethane (BC6)

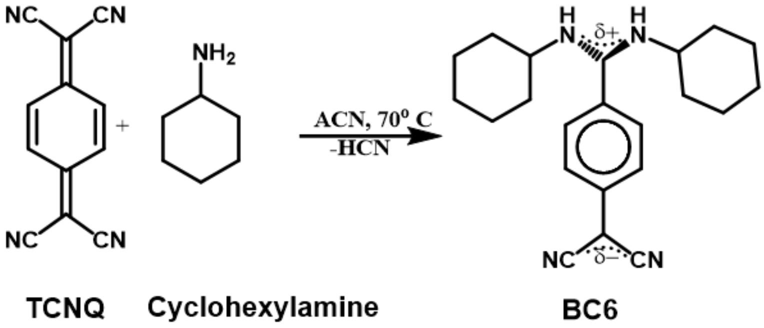

Figure S7. Synthesis of (a) BAP and (b) BC6.

NMR spectroscopic characterization of BAP and BC6

(a) 7,7-bis(2-(2-aminoethy)lpyridino)-8,8-dicyanoquinodimethane (BAP)

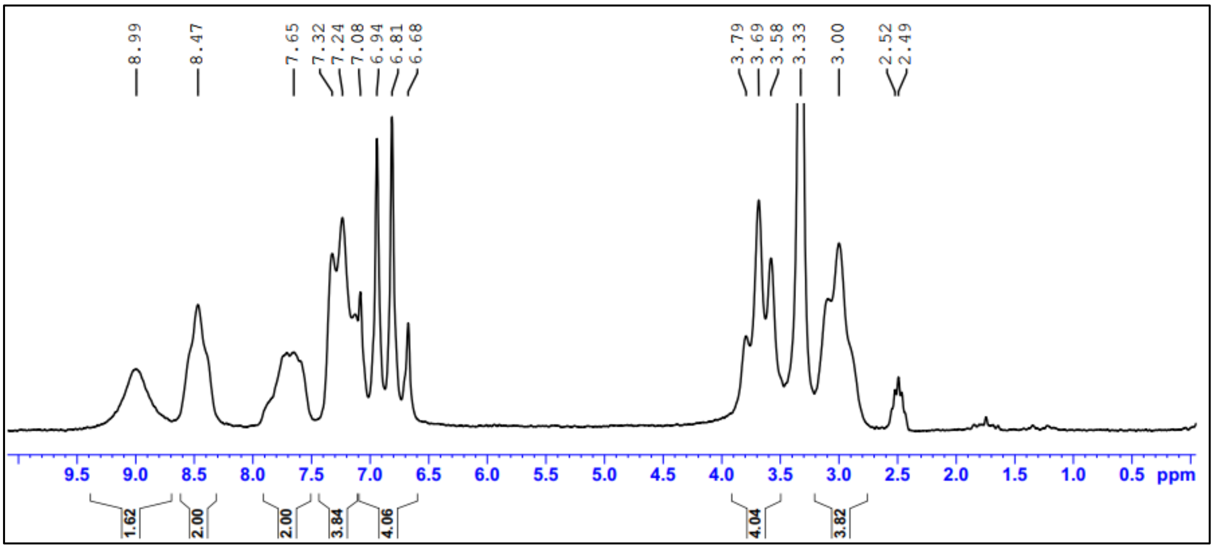

^{1}**H NMR** (60 MHz, DMSO-d_6) δ 8.99 (bs, 2H), δ 8.47 (bs, 2H), δ 7.65 (bs, 2H), δ 7.24 (m, 4H), δ 6.68 (t, J=7.8, 4H), δ 3.58 (t, *J*=6 Hz, 4H), δ 3.00 (m, 4H).

(b) 7,7-bis(cyclohexylamino)-8,8-dicyanoquinodimethane (BC6)

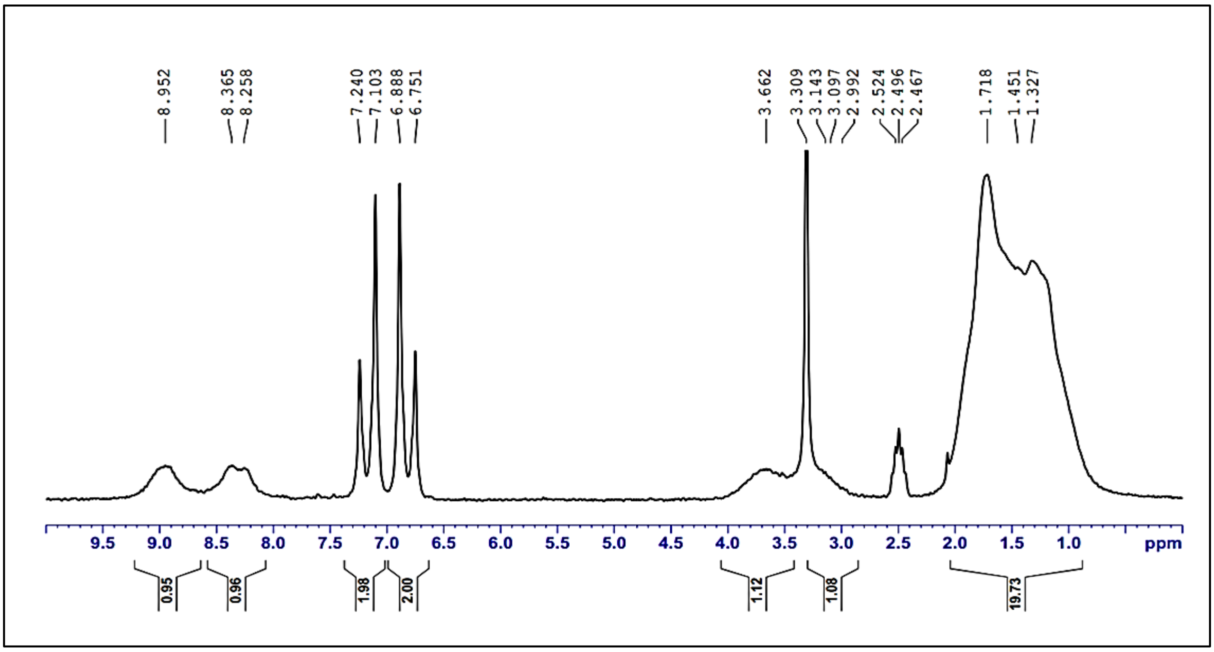

^{1}**H NMR** (60 MHz, DMSO-d_6) δ 8.95 (bs, 1H), δ 8.26 (bs, 1H), δ 7.10 (d, 2H), δ 6.75 (d, 2H), δ 3.66 (m, 1H), δ 2.99 (m, 1H), 1.32 (m, 20H)

Figure S8. ^{1}H NMR spectra of (a) BAP and (b) BC6.

Detection of picric acid

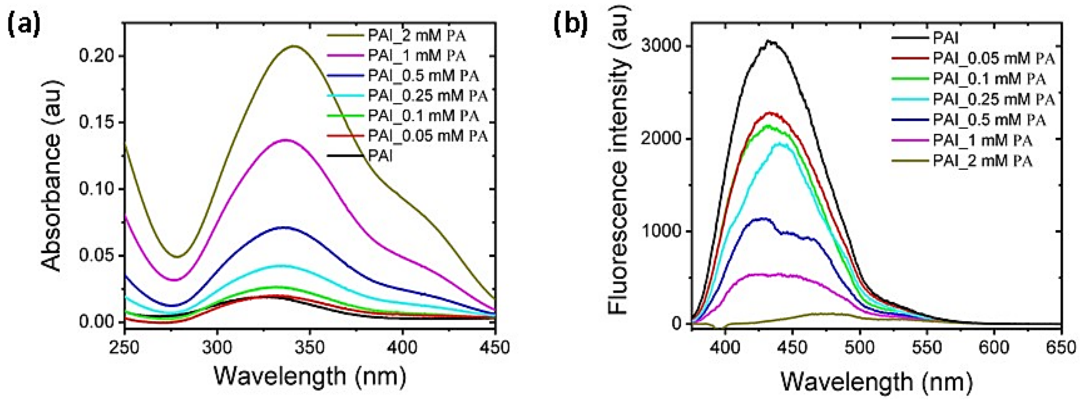

Figure S9. (a) Electronic absorption and (b) fluorescence emission spectra of PAI microrobots with increasing concentration of picric acid (PA).

Molecular structure of nitroaromatic explosives

Caution! The nitroaromatic compounds used in this study (TNT and PA) are highly dangerous explosives. They should be handled very carefully and also in very small quantities.

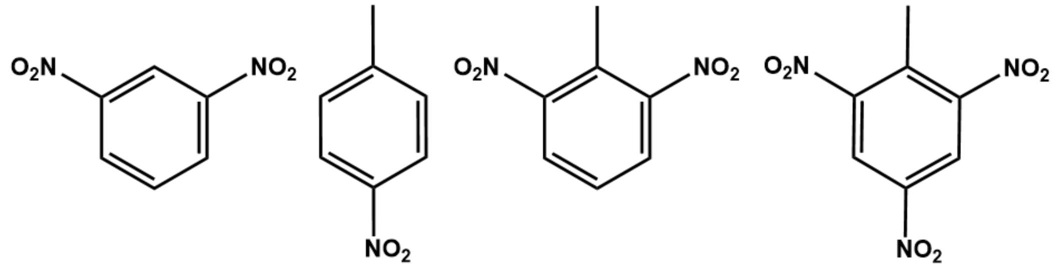

1,3-Dinitrobenzene (DNB) **4-Nitrotoluene (NT)** **2,6-Dinitrotoluene (DNT)** **2,4,6-Trinitrotoluene (TNT)**

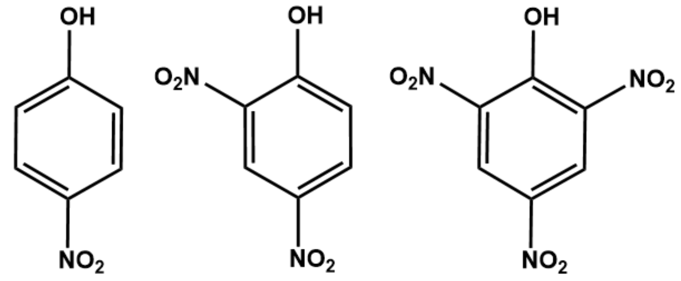

4-Nitrophenol (NPh) **2,4-Dinitrophenol (DNPh)** **Picric acid (PA)**

Figure S10. Molecular structure of nitro compounds used in this study.

Plausible reaction mechanism between PAI and picric acid

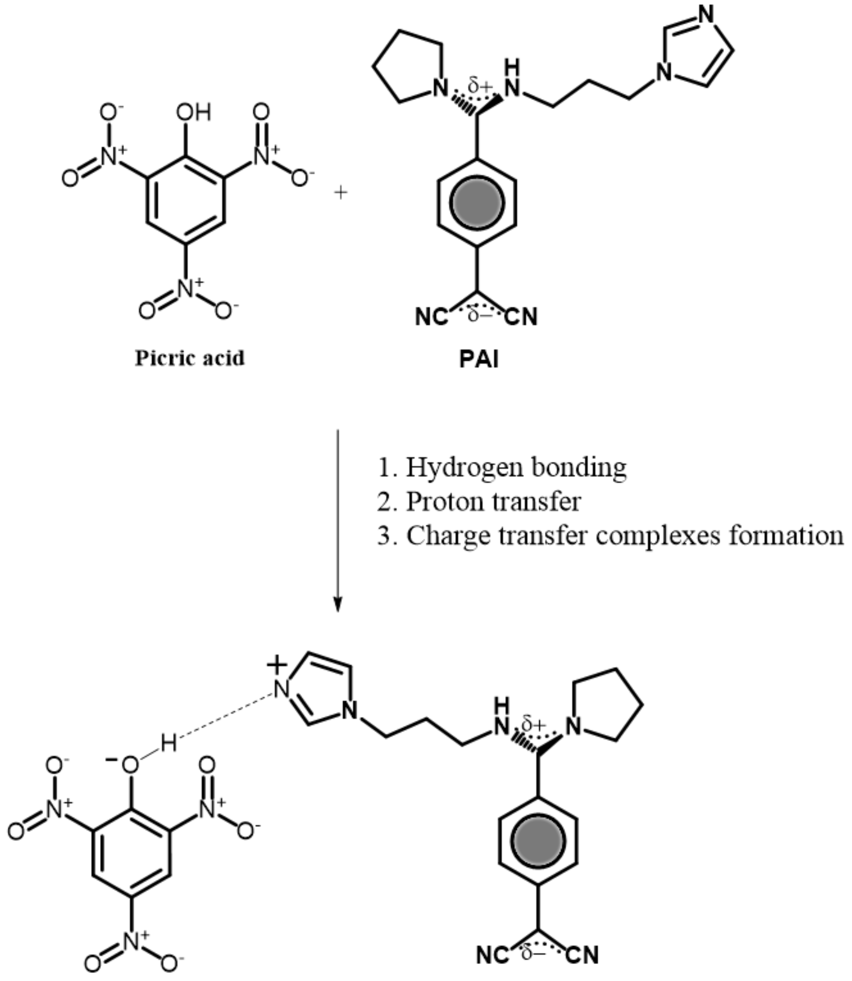

Figure S11. Plausible reaction mechanism between PAI and picric acid (PA).

FTIR spectra of BC6 with picric acid and tetracycline

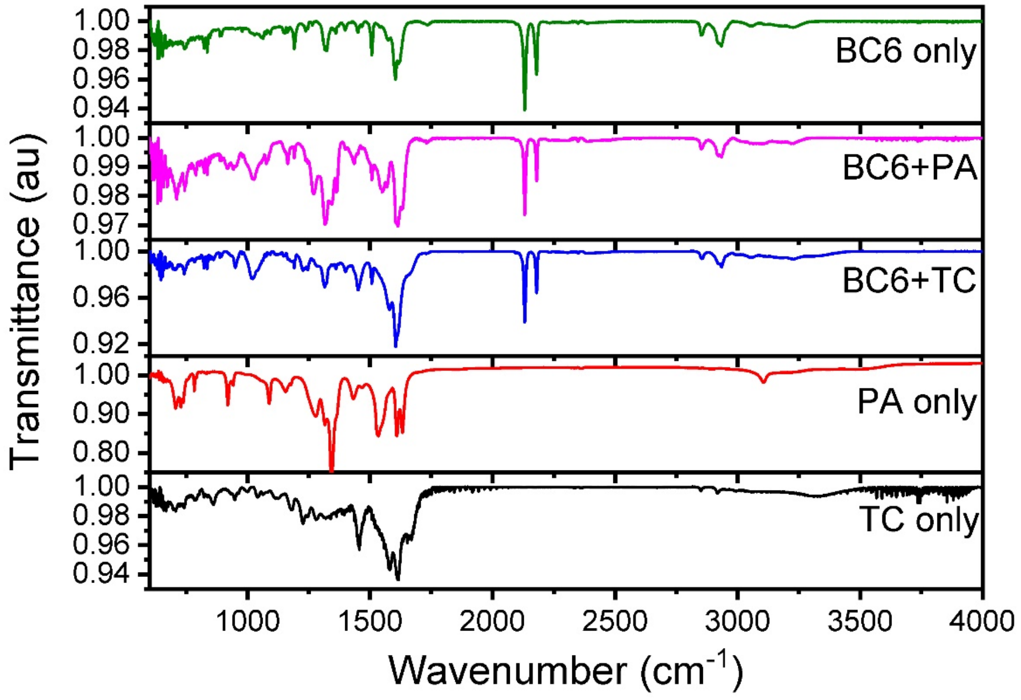

Figure S12. FTIR spectra of bare BC6, BC6 treated with picric acid and tetracycline, bare picric acid (PA), and bare tetracycline (TC).

Limit of detection for picric acid

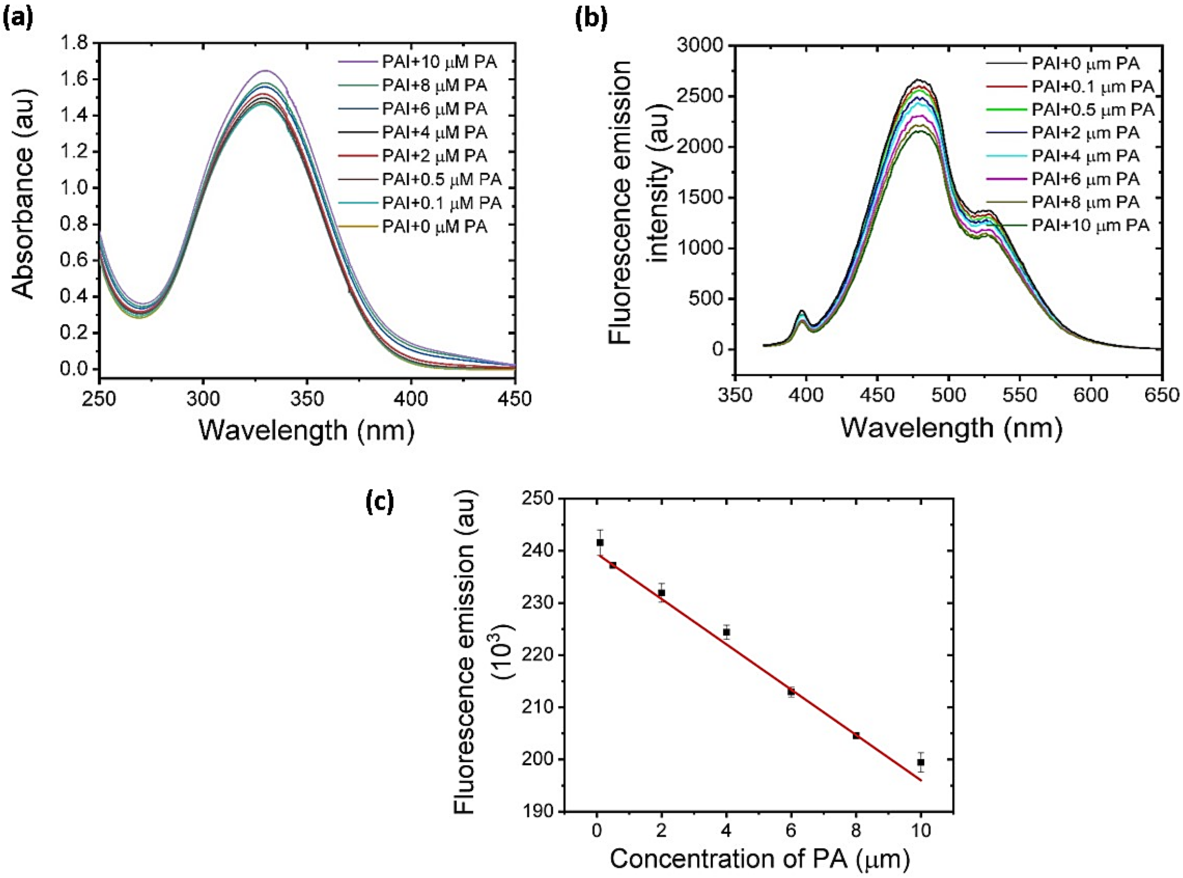

Figure S13. (a) Electronic absorption and (b) fluorescence emission spectra of PAI molecules with increasing concentration of picric acid (PA), (c) Linear plot of fluorescence emission intensity versus concentration of picric acid (PA).

LOD calculation:

Slope (k) = 4344 x 10^6 intensity/M

Standard deviation (σ) = 282 intensity (n=6)

Limit of detection = 3.3 σ/k

= 3.3 x (282 /4344 x 10^6) M

LOD = 0.214 x 10^{-6} M

= 214 nM

Locomotion of microrobots towards dinitrotoluene (DNT)

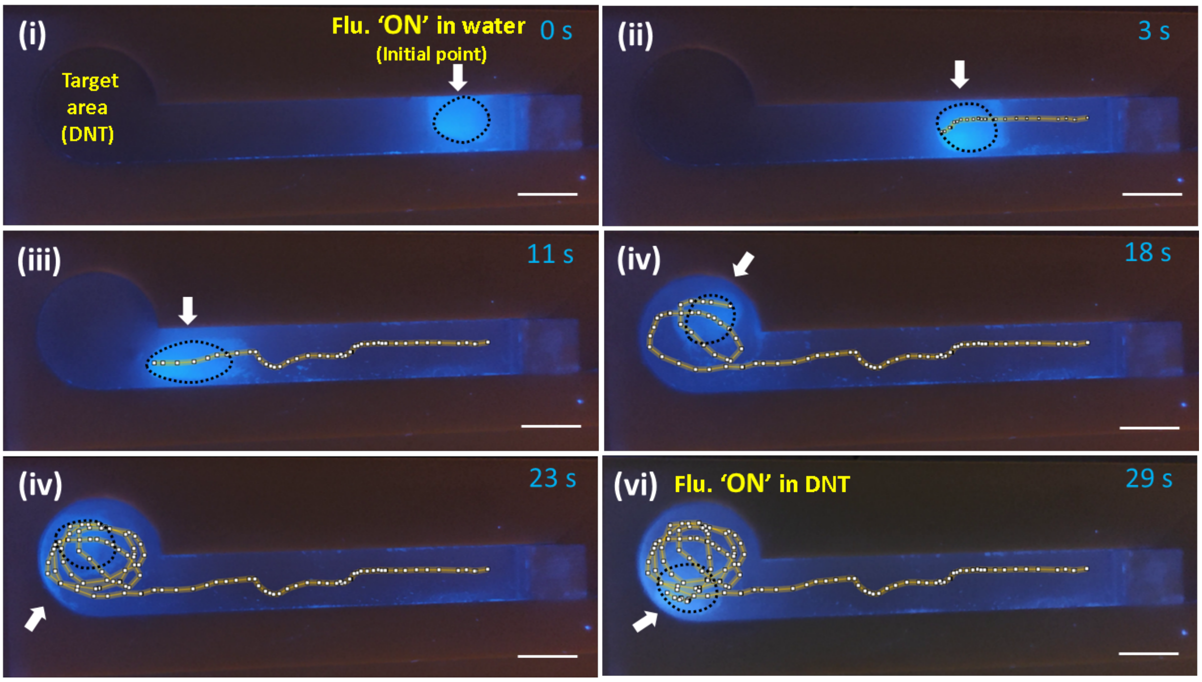

Figure S14. Time lapse locomotion images of microrobots toward the target area filled with dinitrotoluene (DNT) in a fluidic channel (scale bars: 1 cm).

Comparison of detection performance of PAI microrobots with other fluorescent sensing probes to picric acid

Table S2. Comparison of detection performance of various fluorescent probes including MOFs, COFs, small organic molecules, CDs, and polymer materials to picric acid (PA)

Sr. No.	Probes	microrobots	LOD (M)	Solvent system	Ref.
1	Histidine	No	2.71×10^{-6}	Aqueous	S1
2	Anthracene-bridged poly(*N*-vinyl pyrrolidone	No	6×10^{-9}	Aqueous	S2
3	Amine-functionalized α-cyanostilbene derivatives	No	2.85×10^{-7}	Aqueous	S3
4	Quinoline based sensor	No	9.33×10^{-6}	Aqueous	S4
5	Ni-MOF	No	0.29×10^{-6}	Aqueous	S5
6	Porous organic polymer	No	8×10^{-6}	Aqueous	S6
7	Terbium based metal organic framework	No	1.3×10^{-5}	Aqueous	S7
8	Carbon dots-embedded ZIF-8 nanocomposite	No	2.58×10^{-5}	Aqueous	S8
9	Fe_2O_3-CdSe nanocomposite	No	2.2×10^{-6}	Aqueous	S9
10	NP-CQD	No	23×10^{-6}	Aqueous	S10
11	Cd(II) coordination polymer	No	8.36×10^{-6}	Aqueous	S11
12	Aniline-based covalent organic frameworks	No	1.06×10^{-9}	Aqueous	S12
13	Covalent Organic Cage	No	2.7×10^{-9}	Aqueous	S13
14	Nitrogen-Doped Carbon Dots	No	33×10^{-9}	Aqueous	S14
15	PAI	Yes	214×10^{-9}	Aqueous	This work

Detection of Tetracycline

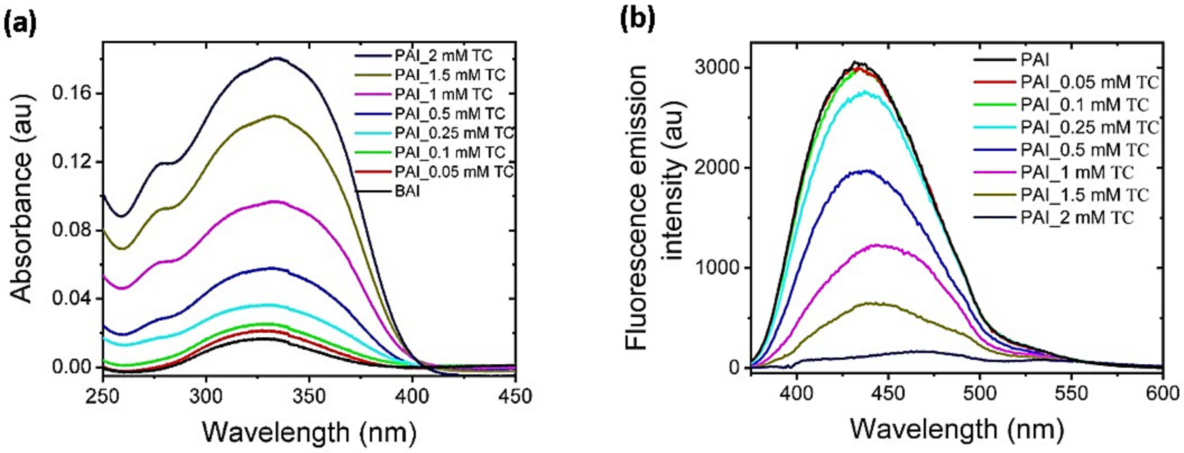

Figure S15. (a) Electronic absorption, and (b) fluorescence emission spectra of PAI microrobots with increasing concentration of Tetracycline (TC).

Plausible reaction mechanism between PAI and tetracycline

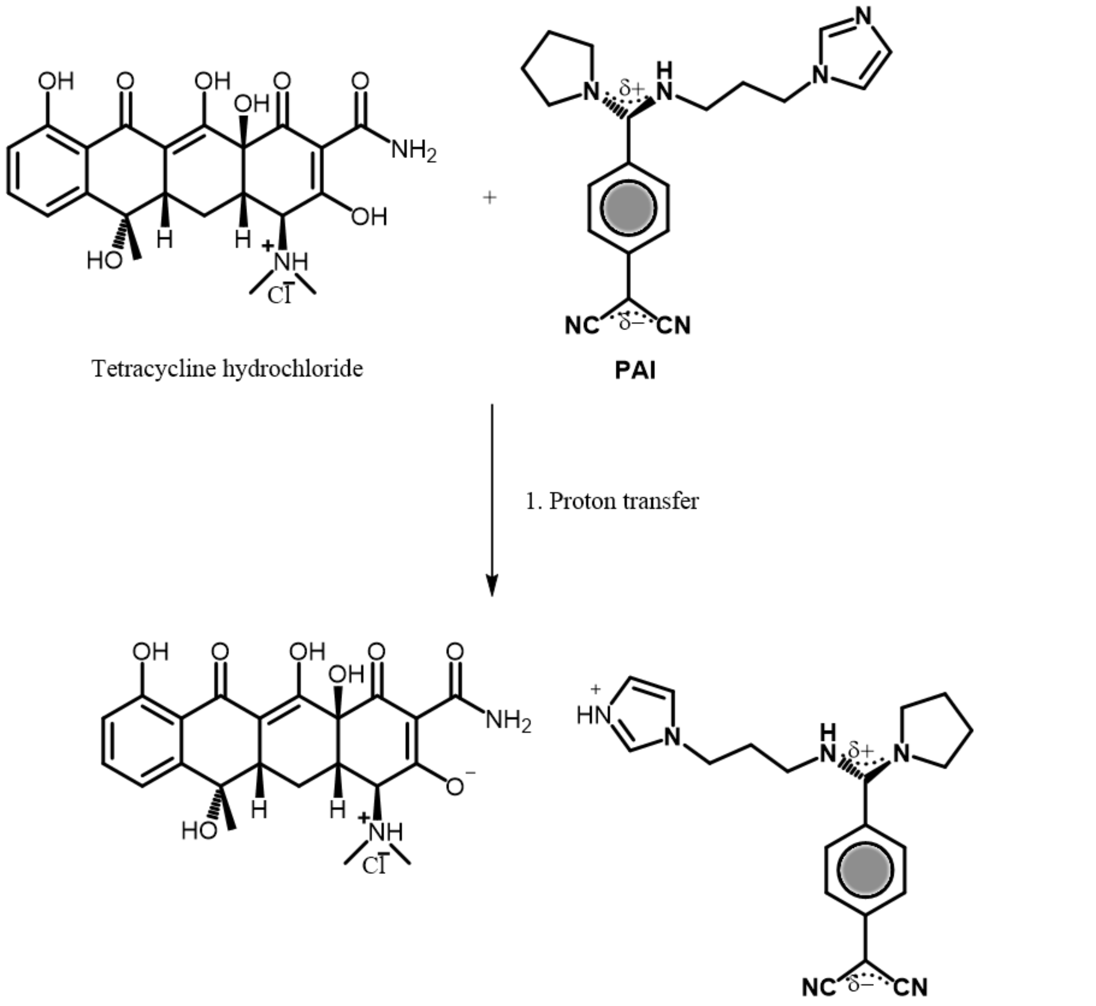

Figure S16. Plausible reaction mechanism between PAI molecule and tetracycline hydrochloride (TC).

Effect of tetracycline on morphology and fluorescence of PAI microrobots

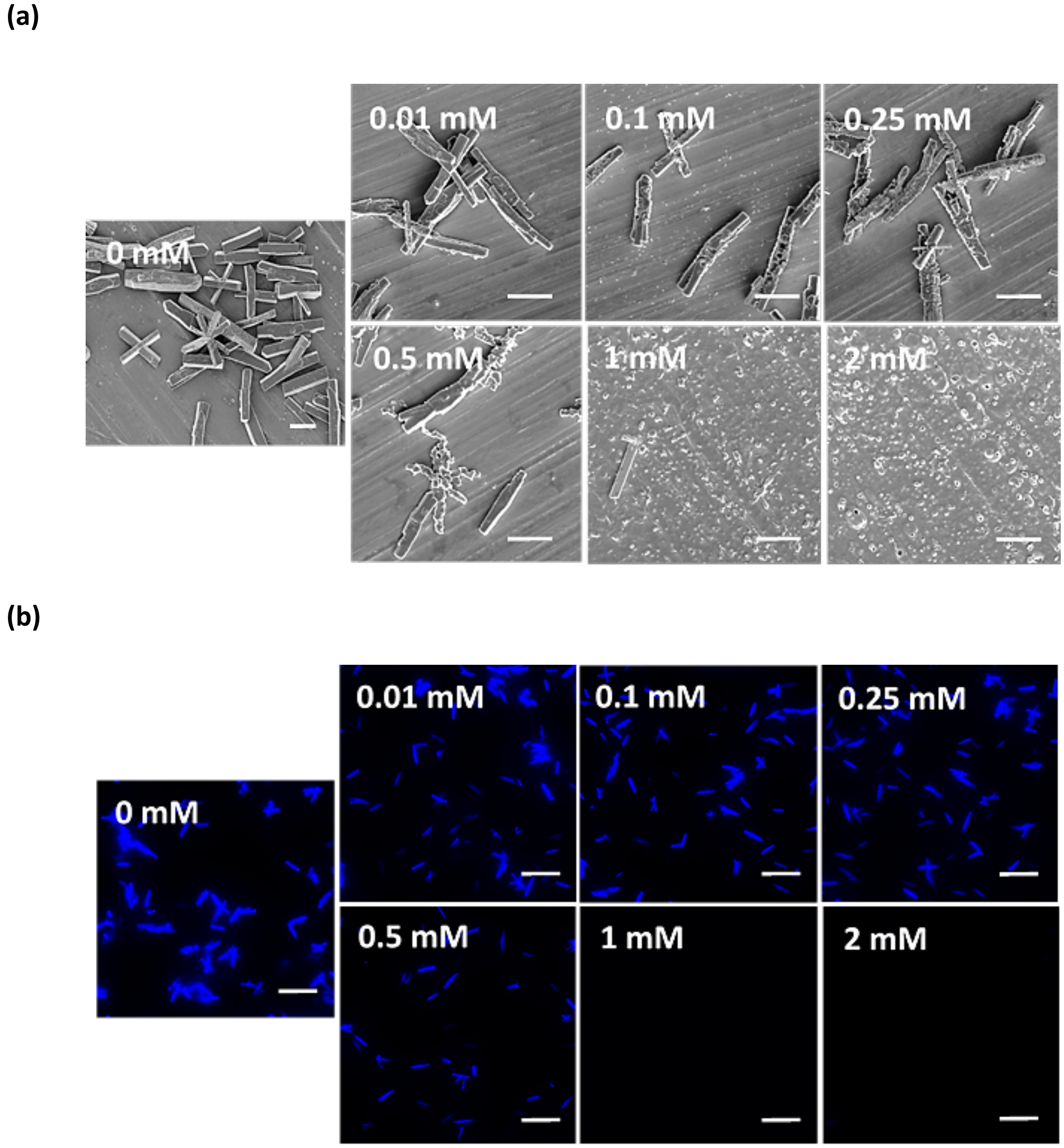

Figure S17. (a) FESEM (scale bars: 10 μm) and (b) fluorescence microscopic images (scale bars: 50 μm) of PAI microrobots upon exposure to increasing concentration of tetracycline (TC).

Limit of detection for Tetracycline

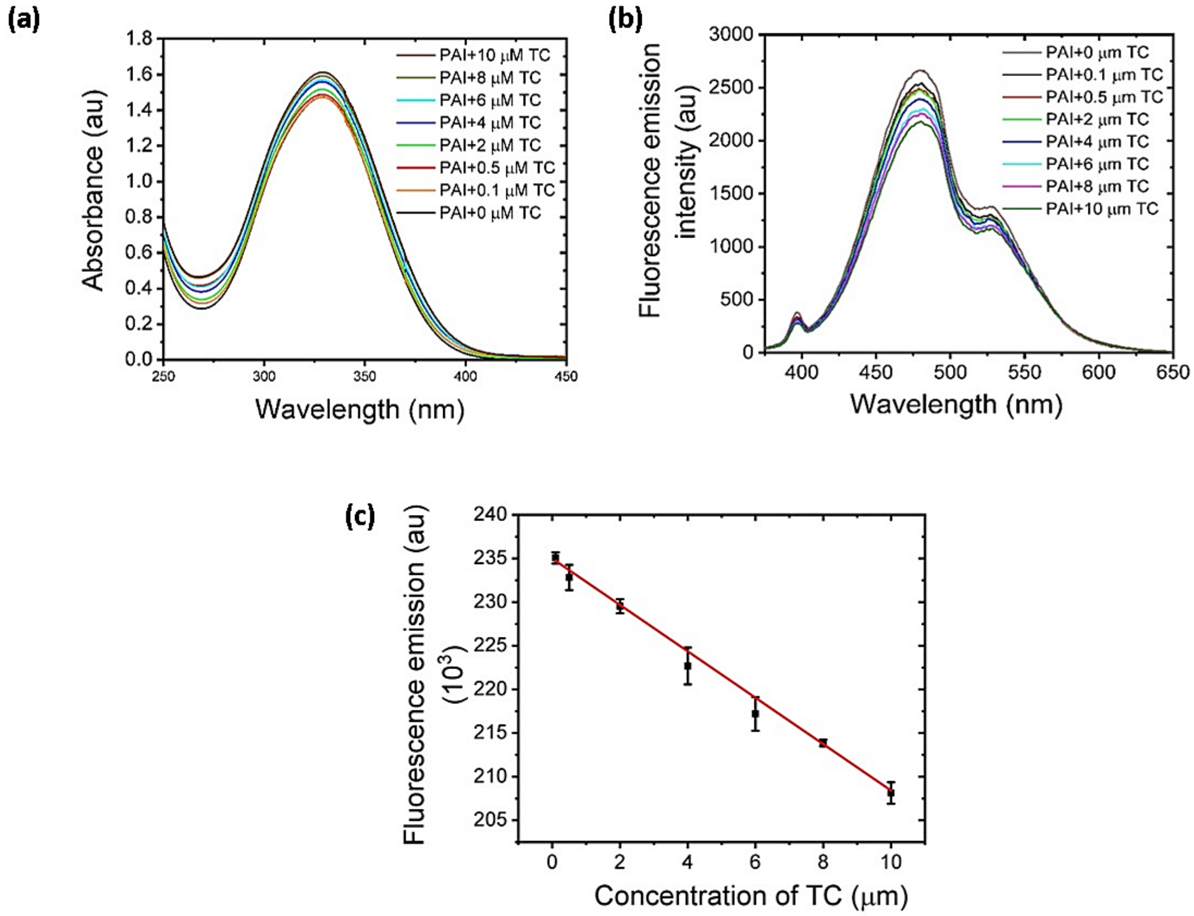

Figure S18. (a) Electronic absorption and (b) fluorescence emission spectra of PAI molecules with increasing concentration of tetracycline (TC). (c) Linear plot of fluorescence emission intensity versus concentration of tetracycline.

LOD calculation:

Slope (k) = 2657 x 10^6 intensity/M

Standard deviation (σ) = 282 intensity (n=6)

Limit of detection = 3.3 σ/k

= 3.3 x (282 / 2657 x 10^6) M

LOD = 0.3502 x 10^{-6} M

= 350 nM

Comparison of detection performance of PAI microrobots with other fluorescent sensing probes to tetracycline

Table S3. Comparison of detection performance of various fluorescent probes including MOFs, DNA aptamer, small organic molecules, and CDs to tetracycline (TC).

Sr. No.	Probes	microrobots	LOD (M)	Solvent system	Ref.
1	Copper doped carbon dots	No	0.16×10^{-6}	Water	S15
2	Self-assembled copper nanoclusters	No	40×10^{-9}	Water, human urine and milk samples	S16
3	Lanthanide coordination polymer nanoparticles	No	3.4×10^{-9}	Water, honey, paper strip	S17
4	4-formyl-3-hydroxybenzoic acid	No	60×10^{-9}	Water, milk and honey samples	S18
5	Hydrogel based on wood-derived cellulose nanocrystals (WCNs) and carbon dots (CDs)	No	0.11×10^{-6}	Aqueous	S19
6	Al-MOF@Mo	No	0.56×10^{-9}	Water, Food samples	S20
7	LMOF-241	No	46 ppb	Aqueous	S21
8	Zeolitic imidazolate framework-8 (ZIF-8)	No	5.99×10^{-6}	water and food samples	S22
9	Glutamic acid–capped iron oxide quantum dots	No	7.69×10^{-9}	Urine	S23
10	Tris-Zn^{2+}	No	$\sim 7.0 \times 10^{-9}$	Water & chicken broth sample	S24
11	ZIF-8	No	14.7×10^{-9}	Aqueous	S25
12	DNA aptamer for oxytetracyline	No	25×10^{-9}	Aqueous	S26
13	UiO-66-NH_2	No	0.449×10^{-6}	Aqueous, milk	S27
14	PAI	Yes	350×10^{-9}	Aqueous	This work

Effect of pH on the detection performance of PAI microrobots to picric acid and tetracycline

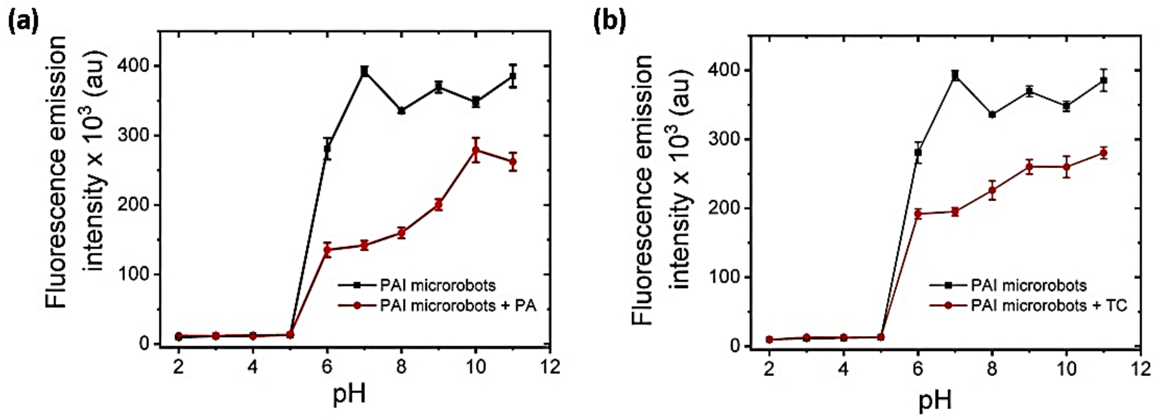

Figure S19. Fluorescence intensity changes of PAI microrobots with (a) picric acid (PA) and (b) tetracycline (TC) in the BR buffer solutions (pH 2-11).

Effect of metal ionic species on the detection performance of PAI microrobots to picric acid

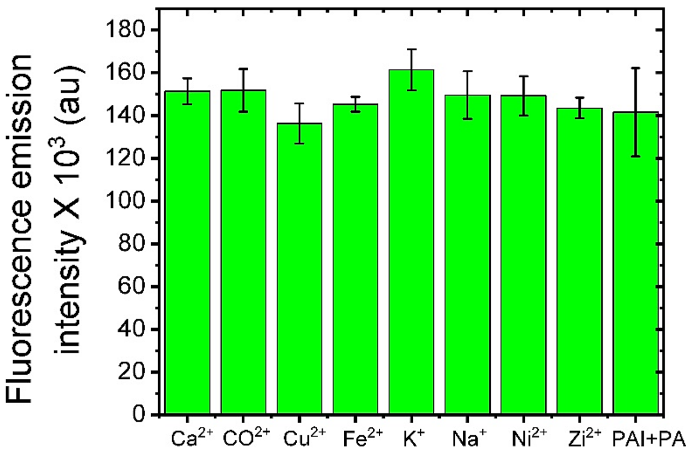

Figure S20. Fluorescence intensity changes of PAI microrobots with picric acid (PA) in various metal ionic species.

References

(S1) Patel, R.; Bothra, S.; Kumar, R.; Sahoo, S. K. Selective Turn-off Sensing of Picric Acid and p-Nitrophenol Using Fluorescent Histidine. *Nano-Structures and Nano-Objects* **2019**, *19*, 100345 (1-5). https://doi.org/10.1016/j.nanoso.2019.100345.

(S2) Singh, R.; Mitra, K.; Singh, S.; Senapati, S.; Patel, V. K.; Vishwakarma, S.; Kumari, A.; Singh, J.; Sen Gupta, S. K.; Misra, N.; Maiti, P.; Ray, B. Highly Selective Fluorescence "turn off" Sensing of Picric Acid and Efficient Cell Labelling by Water-Soluble Luminescent Anthracene-Bridged Poly(: N -Vinyl Pyrrolidone). *Analyst* **2019**, *144* (11), 3620–3634. https://doi.org/10.1039/c8an02417k.

(S3) Ding, A.; Yang, L.; Zhang, Y.; Zhang, G.; Kong, L.; Zhang, X.; Tian, Y.; Tao, X.; Yang, J. Complex-Formation-Enhanced Fluorescence Quenching Effect for Efficient Detection of Picric Acid. *Chem. - A Eur. J.* **2014**, *20* (38), 12215–12222. https://doi.org/10.1002/chem.201402790.

(S4) Sudharsan, S.; Hemalatha, V.; Sarveswari, S.; Vijayakumar, V. A Highly Selective and Sensitive Quinoline-Based Fluorescent Turn-off Chemosensor for the Detection of Picric Acid. *J. Mol. Struct.* **2024**, *1317*, 139087 (1-11). https://doi.org/10.1016/j.molstruc.2024.139087.

(S5) Chongdar, S.; Mondal, U.; Chakraborty, T.; Banerjee, P.; Bhaumik, A. A Ni-MOF as Fluorescent/Electrochemical Dual Probe for Ultrasensitive Detection of Picric Acid from Aqueous Media. *ACS Appl. Mater. Interfaces* **2023**, *15*, 14575−14586. https://doi.org/10.1021/acsami.3c00604.

(S6) Mondal, B.; Das, G. Dual Functional Porous Organic Polymer: Reversible Iodine Capture and Selective Sensing of Picric Acid. *React. Funct. Polym.* **2024**, *194*, 105800 (1-8). https://doi.org/10.1016/j.reactfunctpolym.2023.105800.

(S7) Li, Z. Y.; Yao, Z. Q.; Feng, R.; Sun, M. H.; Shan, X. T.; Su, Z. H.; Li, W.; Bu, X. H. A Highly Stable Terbium Metal-Organic Framework for Efficient Detection of Picric Acid in Water. *Chinese Chem. Lett.* **2021**, *32* (10), 3095–3098. https://doi.org/10.1016/j.cclet.2021.03.008.

(S8) Li, Q.; Chen, S.; Ding, C.; Zhao, S.; Pang, J.; Yan, W. Carbon Dots-Embedded ZIF-8 Nanocomposite for Fluorescence Sensing of Cr(VI) Anion and Picric Acid. *Mater. Lett.* **2024**, *355*, 135464(1-4). https://doi.org/10.1016/j.matlet.2023.135464.

(S9) Kumar, V.; Kumar, A.; Chini, M. K.; Satapathi, S. Fluorescent Fe_2O_3-CdSe Nanocomposite Probe for Selective Detection and Removal of Picric Acid. *Mater. Chem. Phys.* **2021**, *260*, 124130 (1-8). https://doi.org/10.1016/j.matchemphys.2020.124130.

(S10) Babar, D. G.; Garje, S. S. Nitrogen and Phosphorus Co-Doped Carbon Dots for Selective Detection of Nitro Explosives. *ACS Omega* **2020**, *5* (6), 2710–2717. https://doi.org/10.1021/acsomega.9b03234.

(S11) Dou, L.; Tong, L.; Ma, C. Y.; Dong, W. K.; Ding, Y. J. Inserting Auxiliary Ligand to Construct a Cd(II)-Based Salamo-like Coordination Polymer as Bifunctional Chemosensor for Detecting Picric Acid and S2−. *J. Mol. Struct.* **2023**, *1292*, 136162 (1-10). https://doi.org/10.1016/j.molstruc.2023.136162.

(S12) Wang, K.; Geng, T. M.; Zhu, H.; Guo, C. The Preparation of the Flexible Aniline-Based

Covalent Organic Frameworks Used for Uptaking Iodine and Sensing Picric Acid and Iodine. *Microporous Mesoporous Mater.* **2024**, *363*, 112794 (1-10). https://doi.org/10.1016/j.micromeso.2023.112794.

(S13) Mahto, A. K.; Barik, S.; Sarkar, M.; Madda, J. P. A Fluorescent Covalent Organic Cage for Ultrafast Detection of Picric Acid and HCl Vapor Sensing. *Chem. - An Asian J.* **2024**, *20*, e202400912 (1-10). https://doi.org/10.1002/asia.202400912.

(S14) Mahto, M. K.; Samanta, D.; Shaw, M.; Shaik, M. A. S.; Basu, R.; Mondal, I.; Bhattacharya, A.; Pathak, A. Blue-Emissive Nitrogen-Doped Carbon Dots for Picric Acid Detection: Molecular Fluorescence Quenching Mechanism. *ACS Appl. Nano Mater.* **2023**, *6* (9), 8059–8070. https://doi.org/10.1021/acsanm.3c01523.

(S15) Guo, J.; Lu, W.; Zhang, H.; Meng, Y.; Du, F.; Shuang, S.; Dong, C. Copper Doped Carbon Dots as the Multi-Functional Fluorescent Sensing Platform for Tetracyclines and PH. *Sensors Actuators, B Chem.* **2021**, *330*, 129360 (1-8). https://doi.org/10.1016/j.snb.2020.129360.

(S16) Wang, H. B.; Tao, B. B.; Mao, A. L.; Xiao, Z. L.; Liu, Y. M. Self-Assembled Copper Nanoclusters Structure-Dependent Fluorescent Enhancement for Sensitive Determination of Tetracyclines by the Restriction Intramolecular Motion. *Sensors Actuators B Chem.* **2021**, *348*, 130729 (1-8). https://doi.org/10.1016/j.snb.2021.130729.

(S17) Yin, S.; Tong, C. Lanthanide Coordination Polymer Nanoparticles as a Ratiometric Fluorescence Sensor for Real-Time and Visual Detection of Tetracycline by a Smartphone and Test Paper Based on the Analyte-Triggered Antenna Effect and Inner Filter Effect. *Anal. Chim. Acta* **2022**, *1206*, 339809 (1-10). https://doi.org/10.1016/j.aca.2022.339809.

(S18) Sun, P.; Yang, D.; Li, J.; Zhang, Y. Aggregation-Induced Emission of 4-Formyl-3-Hydroxybenzoic Acid for the Ratiometric Fluorescence Detection of Tetracycline Antibiotics. *Dye. Pigment.* **2022**, *197*, 109841 (1-7). https://doi.org/10.1016/j.dyepig.2021.109841.

(S19) Luo, Q.; He, S.; Huang, Y.; Lei, Z.; Qiao, J.; Li, Q.; Xu, D.; Guo, X.; Wu, Y. Non-Toxic Fluorescent Molecularly Imprinted Hydrogel Based on Wood-Derived Cellulose Nanocrystals and Carbon Dots for Efficient Sorption and Sensitive Detection of Tetracycline. *Ind. Crops Prod.* **2022**, *177*, 114528 (1-10). https://doi.org/10.1016/j.indcrop.2022.114528.

(S20) Li, C.; Yang, W.; Zhang, X.; Han, Y.; Tang, W.; Yue, T.; Li, Z. A 3D Hierarchical Dual-Metal-Organic Framework Heterostructure up-Regulating the Pre-Concentration Effect for Ultrasensitive Fluorescence Detection of Tetracycline Antibiotics. *J. Mater. Chem. C* **2020**, *8* (6), 2054–2064. https://doi.org/10.1039/c9tc05941e.

(S21) Hu, Z.; Lustig, W. P.; Zhang, J.; Zheng, C.; Wang, H.; Teat, S. J.; Gong, Q.; Rudd, N. D.; Li, J. Effective Detection of Mycotoxins by a Highly Luminescent Metal-Organic Framework. *J. Am. Chem. Soc.* **2015**, *137* (51), 16209–16215. https://doi.org/10.1021/jacs.5b10308.

(S22) Chen, X.; Xu, J.; Li, Y.; Zhang, L.; Bi, N.; Gou, J.; Zhu, T.; Jia, L. A Novel Intelligently Integrated MOF-Based Ratio Fluorescence Sensor for Ultra-Sensitive Monitoring of TC in Water and Food Samples. *Food Chem.* **2023**, *405*, 134899 (1-9). https://doi.org/10.1016/j.foodchem.2022.134899.

(S23) Sudewi, S.; Zulfajri, M.; Dayalan, S.; Hsu, S. C. N.; Huang, G. G. Glutamic Acid–Capped Iron Oxide Quantum Dots as Fluorescent Nanoprobe for Tetracycline in Urine. *Microchim. Acta* **2023**, *190*, 226 (1-12). https://doi.org/10.1007/s00604-023-05801-3.

(S24) Zhan, Y. C.; Tsai, J. J.; Chen, Y. C. Zinc Ion-Based Switch-on Fluorescence-Sensing Probes for the Detection of Tetracycline. *Molecules* **2022**, *27*, 8403 (1-11). https://doi.org/10.3390/molecules27238403.

(S25) Si, Y.; Li, Y.; Yang, G.; Zhang, S.; Yang, L.; Dai, W.; Wang, H. Zeolitic Imidazolate Framework-8 for Ratiometric Fluorescence Sensing Tetracyclines in Environmental Water Based on AIE Effects. *Anal. Chim. Acta* **2022**, *1199*, 339576 (1-7). https://doi.org/10.1016/j.aca.2022.339576.

(S26) Zhao, Y.; Ong, S.; Chen, Y.; Jimmy Huang, P. J.; Liu, J. Label-Free and Dye-Free Fluorescent Sensing of Tetracyclines Using a Capture-Selected DNA Aptamer. *Anal. Chem.* **2022**, *94* (28), 10175–10182. https://doi.org/10.1021/acs.analchem.2c01561.

(S27) Wang, X.; Wang, X. UiO-66-NH_2 Based Fluorescent Sensing for Detection of Tetracyclines in Milk. *RSC Adv.* **2022**, *12* (36), 23427–23436. https://doi.org/10.1039/d2ra04023a.
